# Inhibition of Macrophage ARID3A Alleviates Myocardial Ischemia‐Reperfusion Injury After Heart Transplantation by Reducing THBS1/CD47 Signaling‐Mediated Neutrophil Extracellular Traps Formation

**DOI:** 10.1002/advs.202509952

**Published:** 2025-09-06

**Authors:** Hao Tian, Yonghong Xiong, Junbiao Zhan, Zhikun Lu, Yuxi Zhang, Yan Leng, Qin Huang, Zhongyuan Xia

**Affiliations:** ^1^ Department of Anesthesiology Renmin Hospital of Wuhan University Wuhan Hubei 430060 P. R. China

**Keywords:** heart transplantation, intercellular crosstalk, ischemia‐reperfusion injury, neutrophil extracellular traps (NETs), single‐cell RNA sequencing

## Abstract

Mitigating myocardial ischemia‐reperfusion (IR) injury is essential for enhancing the success of heart transplantation (HT) and improving patient outcomes. During HT, infiltrating neutrophils are influenced and regulated by various other cell types, contributing to myocardial IR injury through the excessive release of neutrophil extracellular traps (NETs). Nonetheless, the precise mechanisms underlying the interactions between neutrophils and other non‐cardiomyocytes remain largely unexplored. Single‐cell RNA sequencing is employed to characterize the cellular landscape and to explore the crosstalk between neutrophils and other non‐cardiomyocytes. The role of AT‐rich interactive domain‐containing protein 3A (ARID3A) during HT is further examined using myeloid‐specific ARID3A‐knockout mice. Molecular docking analyses are conducted to identify the target of 4‐octyl itaconate (4‐OI). These results reveal that M1 macrophages recruited during the reperfusion of HT promote NETs formation and myocardial IR injury through THBS1/CD47 axis, whereas CD47 induces NETosis by activating the p38 MAPK signaling. Exogenous administration of 4‐OI specifically inhibits ARID3A in macrophages, thereby suppressing NETosis and alleviating myocardial IR injury. These findings indicate that THBS1/CD47 signaling is a critical bridge mediating the interaction between M1 macrophages and NETs‐associated neutrophils, and identify 4‐OI as a promising therapeutic candidate for the treatment of myocardial IR injury following HT.

## Graphical abstract

This study reveals that ARID3A in macrophages can transcriptionally regulate the THBS1‐CD47 axis after heart transplantation (HT), thereby inducing activated neutrophils to release NETs through the p38 MAPK signaling pathway. The itaconate derivative 4‐OI can inhibit ARID3A in macrophages and promote the accumulation of antioxidant factors, thereby alleviating myocardial IR injury and NETosis during HT.

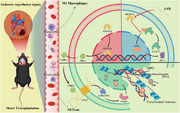

## 1 Introduction

Heart failure has been recognized as a global pandemic, with more than 64 million people estimated to suffer from heart failure worldwide.^[^ ^1^ ^]^ Although various treatment options are available for patients with end‐stage heart failure, heart transplantation (HT) remains the most effective therapeutic intervention.^[^ ^2^ ^]^ However, several complications including IR injury, hyperacute rejection, acute rejection, chronic rejection, and graft vasculopathy have a profound impact on the success of HT.^[^ ^3^ ^]^ While most research to date has concentrated on acute and chronic rejection, IR injury in the context of HT has received comparatively limited attention. Nevertheless, IR injury is a major contributor to early graft failure,^[^ ^4^ ^]^ and it is necessary to elucidate its exact intrinsic molecular mechanisms.

Despite the sterile nature of IR injury, it initiates a complex inflammatory response that critically affects both the extent of cardiac damage and the reparative process. Neutrophil infiltration and accumulation are hallmark features of inflammatory injury following myocardial IR.^[^ ^5^ ^]^ As frontline responders in the innate immune response, neutrophils are rapidly activated during myocardial IR and are recruited to the injured myocardium in large numbers in response to chemokines released at the site of damage.^[^ ^6^ ^]^ Emerging evidence suggests that neutrophils exacerbate IR injury in transplanted organs through a highly efficient mechanism.^[^ ^7^ ^]^ Specifically, some neutrophils release a web‐like structure composed of decondensed chromatin and antimicrobial proteins, including histones, cytoplasmic enzymes, proteases, and granule components, known as neutrophil extracellular traps (NETs). The process by which neutrophils form NETs is called NETosis.^[^ ^8^ ^]^ Traditionally, NETs have been viewed as beneficial, serving to trap and eliminate pathogens, attenuate inflammatory responses, and maintain the health of the organism.^[^ ^9^ ^]^ With the deepening of related studies, more and more evidence suggest that excessive formation and insufficient clearance of NETs can contribute to the pathogenesis and progression of various diseases, including inflammatory disorders, autoimmune diseases, thrombosis‐related conditions, and malignancies.^[^ ^10^ ^]^ Although NETs have recently garnered considerable attention for their role in cardiovascular disease,^[^ ^11^ ^]^ their contribution to myocardial IR injury after HT and the associated molecular mechanisms remain incompletely elucidated.

It has been demonstrated in recent studies that inhibition of macrophage‐associated mediator activation shows great potential in limiting NETosis‐related injury.^[^ ^12^ ^]^ Within the immune microenvironment of the heart, macrophages represent the most abundant population of immune cells,^[^ ^13^ ^]^ constituting ≈6–8% of non‐cardiomyocytes in adult mice.^[^ ^14^ ^]^ Based on their activation states and functional roles, macrophages are broadly categorized into classically activated (M1) and alternatively activated (M2) phenotypes. During myocardial IR injury, circulating monocytes infiltrate the myocardium and predominantly differentiate into M1 macrophages. These M1 macrophages release a variety of pro‐inflammatory cytokines, such as tumor necrosis factor‐α (TNF‐α) and interleukin‐1β (IL‐1β), which amplify the inflammatory response.^[^ ^15^ ^]^ Emerging evidence has validated the therapeutic promise of targeted modulation of M1 macrophage polarization in alleviating IR injury during HT.^[^ ^16^ ^]^ Therefore, elucidating the molecular mechanisms by which M1 macrophages involved in myocardial IR injury could provide critical insights for developing clinical therapies to enhance graft survival in HT.

Cell‐cell interactions between different cell populations during reperfusion in HT are essential to exacerbate myocardial IR injury. Following ischemic injury, the cellular composition of the heart undergoes rapid changes, with dynamic interactions occurring among neutrophils, macrophages, fibroblasts, endothelial cells, epicardial cells, and cardiomyocytes to form a complex, spatiotemporally regulated cardiac interactome.^[^ ^17^ ^]^ For instance, cardiomyocytes subjected to IR stress release small extracellular vesicles containing mitochondrial DNA (mtDNA), which in turn activate and promote the proliferation of fibroblasts.^[^ ^18^ ^]^ Additionally, phosphodiesterase 4B (PDE4B), highly expressed in myeloid and endothelial cells after myocardial reperfusion, has been shown to mediate neutrophil‐driven inflammation, impair coronary microvascular perfusion, and aggravate myocardial IR injury.^[^ ^19^ ^]^ Other studies have identified key molecular regulators involved in macrophage‐fibroblast communication that contribute to pathological fibroblast activation.^[^ ^20^ ^]^ Despite these advances, the mechanisms underlying the interaction between macrophages and neutrophils, which are two key players in the innate immune response, remain incompletely understood. Taken together, considering the important role of NETs in myocardial IR injury with HT and their undefined role in relation to macrophages, the present study focuses on the molecular mechanisms by which macrophages promote NETosis formation during the progression of reperfusion injury in HT.

Single‐cell RNA sequencing (scRNA‐seq) has emerged as a powerful tool for deciphering complex cellular communications and associated biological processes, offering unprecedented resolution for mapping cell‐cell interactions within the transplanted heart microenvironment.^[^ ^21^ ^]^ In our investigation, we identified and characterized a neutrophil subpopulation associated with NETs in the hearts of mice with HT‐induced IR injury by scRNA‐seq. Furthermore, we confirmed that thrombospondin‐1 (THBS1)/CD47 is a key ligand‐receptor pair for M1 macrophage‐induced NETosis. AT‐rich interactive domain‐containing protein 3A (ARID3A), a transcription factor from the ARID family, was found to be upregulated in pro‐inflammatory macrophages.^[^ ^22^ ^]^ By co‐culturing with hypoxia‐reoxygenation (H/R) solutions induced by the cardiomyocyte H/R model, the present study revealed that ARID3A transcriptionally regulates THBS1 secretion from M1 macrophages. Moreover, myeloid‐specific ARID3A‐deficient mice exhibited significantly ameliorated myocardial injury. As an endogenous metabolite, itaconate offers superior biocompatibility and safety compared to many synthetic drugs,^[^ ^23^ ^]^ and both itaconate and its derivatives have shown promise in cardiovascular research.^[^ ^24^ ^]^ Many studies on itaconate have used derivative compounds 4‐octyl itaconate (4‐OI) because of the greater cell permeability of 4‐OI compared to itaconate itself, its thiol reactivity similar to that of itaconate, and its ability to be converted to itaconate intracellularly.^[^ ^25^ ^]^ Significantly, we found that exogenous administration of 4‐OI could target the inhibition of ARID3A in macrophages, thus exerting a therapeutic effect following IR injury after HT. In conclusion, this study not only provides new insights into the pathomechanism of myocardial IR injury in HT, but also offers a theoretical basis for the development of NETs‐targeted therapeutic strategies.

## 2 Results

### 2.1 NETosis is Involved in Myocardial IR Injury After HT

**Figure 1:**
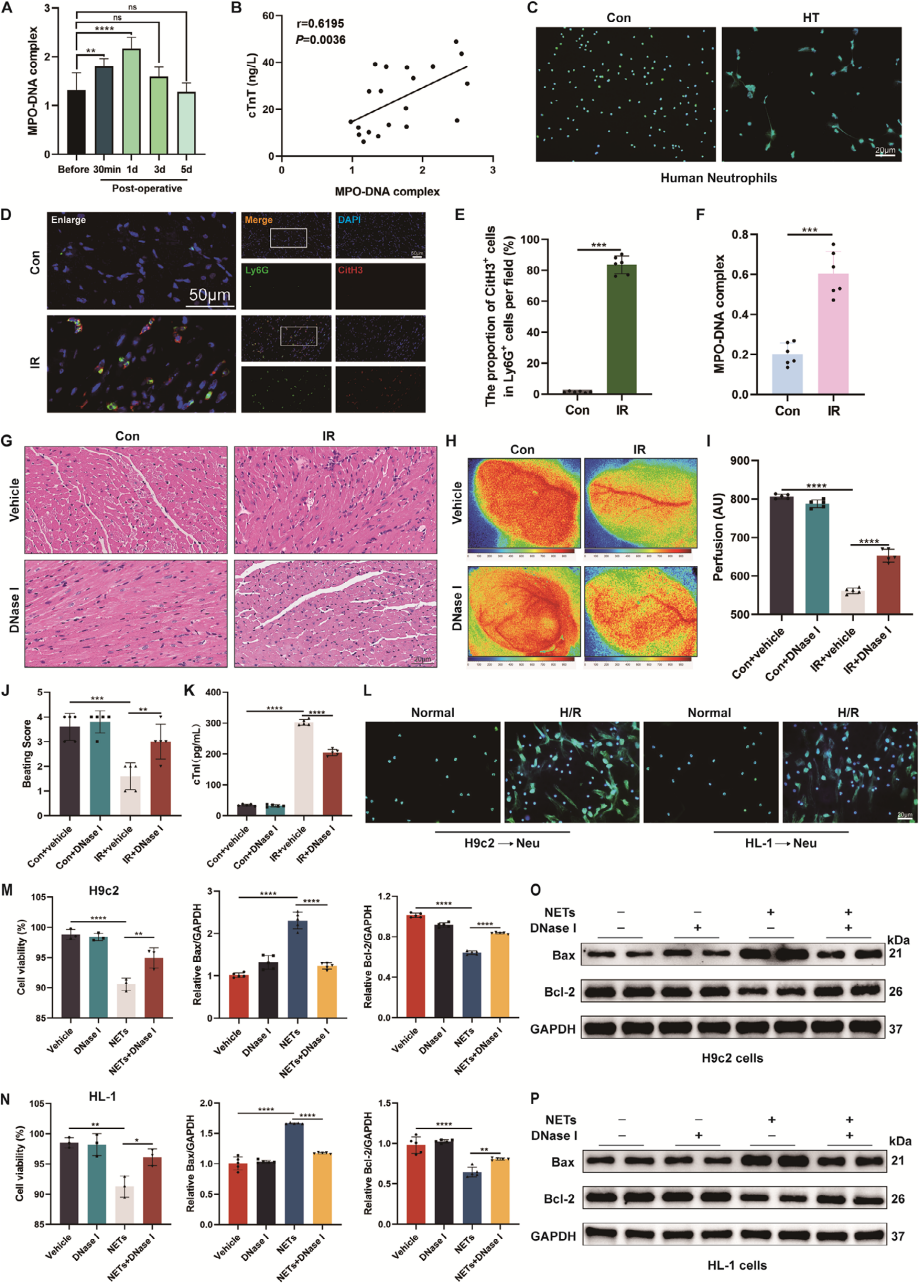
NETs are involved in the occurrence and development of IR injury during HT. A) Quantitative analysis of MPO‐DNA complex in serum of patients undergoing HT during the perioperative period (*n* = 7). B) The Spearman correlation analysis of cTnT and MPO‐DNA complex in serum (*n* = 20). C) Spontaneous NETs formation of human peripheral blood neutrophils was measured using SYTOX Green staining. Scale bar = 20 µm. D, E) Representative immunofluorescence images and quantification of Ly6G^+^ CitH3^+^ double‐positive cells with staining for Ly6G (green) and CitH3 (red) in heart tissues (*n* = 6). Scale bar = 50 µm. F) Quantitative analysis of MPO‐DNA complex in serum of mice (*n* = 6). G) Representative image of H&E staining of heart tissues. Scale bar = 20 µm. H, I) Representative peak perfusion images in arbitrary units (AU) and statistical analysis of mean perfusion (*n* = 5). Pseudo‐colored scale bar represents level of perfusion with higher values in red and lower values in blue. J) Beating score measured by the Stanford Cardiac Surgery Laboratory graft scoring system (*n* = 5). K) The serum levels of cTnI were evaluated in different groups of mice (*n* = 5). L) SYTOX Green staining was used to measure the morphology and levels of NETs in human peripheral blood neutrophils co‐cultured with H9c2 and HL‐1 cells derived H/R supernatants (*n* = 6). Scale bar = 20 µm. M, N) Cell viability of H9c2 and HL‐1 cells in different groups (*n* = 3). O, P) The levels of Bax and Bcl‐2 proteins in H9c2 and HL‐1 cells were measured by western blotting (*n* = 5). ns: No significant, ^*^ *p* <0.05, ^**^ *p* <0.01, ^***^ *p* <0.001, ^****^ *p* <0.0001.

To investigate the changes of serum NETs in HT patients, we collected blood from heart transplant patients during the perioperative period. By detecting circulating myeloperoxidase‐DNA (MPO‐DNA) complex levels, it was found that circulating NETs levels began to increase significantly after HT compared with before surgery, peaked at 24 h, and then gradually returned to baseline levels within 5 d (**Figure** **1**A). Furthermore, our correlation analysis of serum cardiac troponin T (cTnT) levels and MPO‐DNA complex levels showed a positive correlation between the levels of NETs and myocardial injury in patients with HT, suggesting that NETs are associated with the development of myocardial IR injury (Figure 1B). In addition, we isolated human peripheral blood neutrophils, with neutrophil purity validated by flow cytometry and Wright‐Giemsa staining (Figure S1B–D, Supporting Information). Peripheral blood neutrophils from healthy volunteers and HT recipients were fluorescently stained using SYTOX Green. Our findings revealed that patients with HT had a markedly higher rate of spontaneous NETs production than healthy individuals as seen in Figure 1C. These clinical evidence suggest that neutrophils are more sensitive to be induced to produce NETs in HT patients compared to healthy volunteers and that NETosis is strongly associated with the development of myocardial IR injury in HT patients.

To better simulate the pathophysiology of reperfusion injury in HT, we constructed a mouse cervical ectopic HT model (Figure S1A, Supporting Information). We examined the degree of neutrophil infiltration and the level of NETs in the donor heart at multiple time points between 2 and 48 h after HT. Consistent with previous reports, we observed that neutrophils were rapidly recruited to heart grafts ≈2 h following reperfusion and the density of infiltrating neutrophils reached maximum after 24 h (Figure S1E, Supporting Information).^[^ ^26^ ^]^ Notably, untransplanted donor hearts examined under similar conditions displayed negligible neutrophil presence, suggesting that the neutrophils recruited after myocardial IR injury almost exclusively originate from the recipient circulation (Figure S1E, Supporting Information). By examining the protein levels of citrullinated histone H3 (CitH3), a marker of neutrophil NETs, in the peripheral blood of mice, we found that NETs levels were highest at 24 h after reperfusion of HT (Figure S1F, Supporting Information). Based on the temporal concordance of neutrophil infiltration and NETs formation, we selected the 24 h time point post‐HT reperfusion for subsequent experimental investigations.

We first sought to determine whether NETs could be identified in myocardium undergoing IR injury. To observe NETs in myocardial tissues, immunofluorescence staining was performed using fluorescent labeling of Ly6G and CitH3, and the occurrence of NETs was determined based on the co‐localization of these factors. The results showed that CitH3 fluorescence expression was almost undetectable in the hearts of control mice, whereas the fluorescence intensity of CitH3 was significantly increased 24 h after IR injury (Figure 1D,E). The level of MPO‐DNA complex, one of the biomarkers of NETosis, is closely associated with the generation of NETs.^[^ ^27^ ^]^ Therefore, to detect the levels of NETs induced by reperfusion injury in HT, we quantified the circulating levels of MPO‐DNA complex in two groups of mice. The results showed that the circulating levels of MPO‐DNA complex were significantly increased in the IR group of mice compared with the control group (Figure 1F). To verify the involvement of NETosis in the pathogenesis of myocardial IR injury in HT, we investigated whether inhibition of NETs would ameliorate cardiac injury in mice after HT. Hematoxylin and eosin (H&E) staining of heart tissues revealed that the IR group showed obvious pathological features of myocardial destruction compared with the control group, with deformation of myocardial fibers, swelling and rupture of cardiomyocytes, and obvious inflammatory cell infiltration (Figure 1G). Speckle tomography of cardiac blood flow showed a weak hemorheological signal in transplanted hearts of IR group (Figure 1H,I). And mice in the IR group exhibited lower beating scores and higher circulating cardiac troponin I (cTnI) levels than the control group (Figure 1J,K). Subsequently, we treated mice with deoxyribonuclease I (DNase I), which is recognized as an inhibitor of NETs formation by degrading chromatin.^[^ ^28^ ^]^ Compared with the vehicle group, the administration of DNase I effectively attenuated myocardial IR injury, increased beating scores, and increased myocardial blood perfusion in the transplanted myocardium (Figure 1G–K).

Given the elevated NETs levels observed in HT patients and murine models after HT, we next aimed to validate these findings in vitro. We established the H/R model of cardiomyocytes, collected the supernatants from cells after reoxygenation (referred to as H/R supernatants), and co‐cultured H/R supernatants with isolated human peripheral blood neutrophils for 6 h (Figure S1G, Supporting Information). Then SYTOX Green staining was carried out to detect the formation of NETs, and the results showed that H/R supernatants produced by both H9c2 and HL‐1 cells could induce the formation of NETs (Figure 1L). Compared with atrial HL‐1 cells, ventricular H9c2 cells are energetically more similar to primary cardiomyocytes, and H9c2 cells have been widely used as an in vitro model to simulate myocardial IR injury.^[^ ^29^ ^]^ Therefore, our subsequent experiments used H9c2 cardiomyocyte‐derived H/R supernatants to simulate the microenvironment after IR injury in transplanted hearts. In summary, the above results fully demonstrate that NETosis is involved in the occurrence and development of myocardial IR injury after HT.

Other studies have confirmed that the presence of NETs causes damage to surrounding tissues and cells.^[^ ^30^ ^]^ To further explore whether NETs induce damage in cardiomyocytes, we isolated neutrophils from fresh blood of healthy volunteers, induced them to produce NETs in vitro, and purified the NETs through a series of steps. Purified NETs were co‐cultured with H9c2 or HL‐1 cells, with or without DNase I. NETs exposure resulted in a significant, dose‐dependent reduction in cardiomyocyte viability. Treatment with 200 or 400 ng mL^−1^ of NETs significantly decreased viability in both cell lines (Figure S1H,I, Supporting Information), and these concentrations were used in subsequent experiments. Cell Counting Kit‐8 (CCK‐8) assays and western blotting consistently demonstrated that NETs induced notable cardiomyocytes injury, whereas DNase I treatment mitigated this effect, as evidenced by enhanced cell viability and reduced apoptosis (Figure 1M–P). Collectively, these findings indicate that neutrophil‐derived NETs are key mediators of cardiomyocyte injury during reperfusion following HT. Targeting NETs formation thus represents a promising therapeutic strategy for the prevention and treatment of myocardial IR injury in HT recipients.

### 2.2 Heterogeneity and Functional Characterization of Myocardial Neutrophils After HT Revealed by scRNA‐Seq

**Figure 2:**
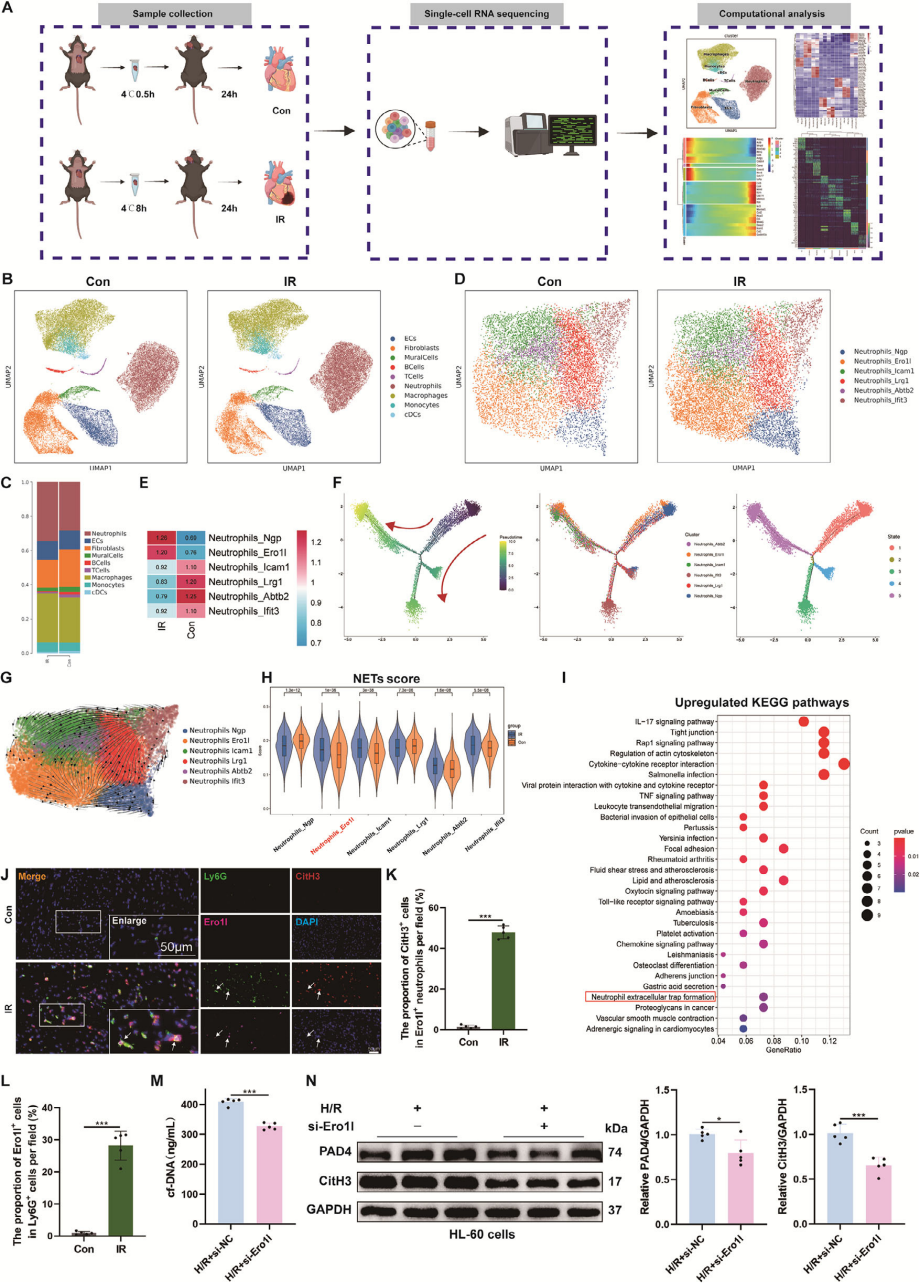
ScRNA‐seq reveals a NETs‐associated neutrophil subset in the hearts of mice undergoing HT. A) Graphical overview of this single‐cell sequencing study. The hearts of mice were dissociated into single‐cell suspensions, and non‐cardiomyocytes were analyzed for scRNA‐seq with 10 × Genomics, followed by comprehensive analysis. B) UMAP analysis of 36835 cells from control hearts and 36704 cells from IR hearts was performed, with 9 clusters shown in each plot (*n* = 3). C) Barplot presenting the proportion of all cell types in each group. D) UMAP plot of neutrophil sub‐clusters in each group. E) Ro/e analysis assessed the percentage difference in each neutrophil cluster between two groups. F) The left pseudotime trajectory is shown colored in a gradient from dark blue to yellow and the start of pseudotime is indicated. The middle pseudotime trajectory is shown colored by different neutrophil sub‐clusters. The right pseudotime trajectory is shown colored by different cell states. G) RNA velocity of neutrophil sub‐clusters. H) Violin plots showing NETs score for each neutrophil cluster. I) KEGG pathway enrichment analysis of up‐regulated differential genes in Ero1l^+^ neutrophil subpopulation, which was significantly enriched in the NETs pathway. J) Immunofluorescence co‐staining for Ly6G (green), CitH3 (red), and Ero1l (magenta) in the hearts of mice from Con and IR groups. Scale bar = 50 µm. K, L) Quantification analysis of Ly6G^+^ Ero1l^+^ cells and Ly6G^+^ Ero1l^+^ CitH3^+^ cells (*n* = 5). M) The levels of cell‐free DNA (cfDNA) in the supernatants of HL‐60 cells co‐cultured with H9c2‐derived H/R supernatants (*n* = 5). N) The levels of PAD4 and CitH3 proteins in HL‐60 cells were measured by western blotting (*n* = 5). ns: No significant, ^*^ *p* <0.05, ^**^ *p* <0.01, ^***^ *p* <0.001, ^****^ *p* <0.0001.

Given the variations of different cell type compositions within the heart, NETs may arise due to alterations in complex intercellular communication. Herein, we sorted cardiac non‐cardiomyocytes from transplanted hearts to perform scRNA‐seq (**Figure** **2**A). Our data revealed cellular diversity in transplanted hearts, identifying a total of 9 cell clusters attributed to putative biological identities based on differentially expressed genes (DEGs) (Figure S2A,B, Supporting Information). Fibroblasts, monocytes, macrophages, endothelial cells (ECs), mural cells, neutrophils, T cells, B cells, and conventional dendritic cells (cDCs) were identified (Figure 2B). Consistent with previous results, we observed a significant increase in the proportion of neutrophils in hearts undergoing IR after HT (Figure 2C).

To further investigate the heterogeneity and functional characteristics of neutrophils, we performed unsupervised clustering of the neutrophil population. According to typical marker expression, a total of 6 subclusters, namely Ngp^+^ neutrophils, Ero1l^+^ neutrophils, Icam1^+^ neutrophils, Lrg1^+^ neutrophils, Abtb2^+^ neutrophils, and Ifit3^+^ neutrophils, appeared within the neutrophil lineages (Figure 2D; Figure S2C,D, Supporting Information). Notably, we found the proportions of Ngp^+^ neutrophils and Ero1l^+^ neutrophils were significantly increased in IR samples compared with controls (Figure 2E; Figure S3A, Supporting Information). This phenomenon suggests that Ngp^+^ neutrophils and Ero1l^+^ neutrophils subsets might play a pivotal role in HT. We then used RNA velocity and pseudotemporal trajectories to reveal the developmental trajectory of neutrophils. These analyses revealed that Ero1l^+^ neutrophils are at the end of differentiation (Figure 2F,G). The reported study indicated that mature neutrophils are more likely to produce NETs compared to undifferentiated neutrophils.^[^ ^31^ ^]^ Therefore, we performed the NETs score on Ero1l^+^ neutrophils based on 69 NETs‐associated genes (Figure 2H; Table S1, Supporting Information).^[^ ^32^ ^]^ NETs‐associated genes in Ero1l^+^ neutrophils were significantly activated (Figure S3B,C, Supporting Information). Based on the above evidence, we defined Ero1l^+^ neutrophils as “NETs‐related neutrophils”. Gene ontology (GO) enrichment analysis suggested that genes highly expressed in Ero1l^+^ neutrophils were mostly the genes involved in neutrophil migration, leukocyte chemotaxis, and regulation of the inflammatory response, suggesting that the activation of these processes is associated with Ero1l^+^ neutrophils (Figure S3D, Supporting Information). In addition, Ero1l^+^ neutrophils showed increased neutrophil chemotaxis, chemokine production, pro‐inflammatory and inflammatory response, confirming the pro‐inflammatory phenotype of Ero1l^+^ neutrophils (Figure S3E–H, Supporting Information). By performing Kyoto Encyclopedia of Genes and Genomes (KEGG) pathway analysis on the up‐regulated genes between control and IR, we found that Ero1l^+^ neutrophils were related to Neutrophil extracellular trap formation (Figure 2I), indicating a critical role for NETs in the subpopulation of Ero1l^+^ neutrophils involved in myocardial IR injury. Immunofluorescence staining confirmed the presence of a neutrophil population with high endoplasmic reticulum oxidoreductase 1 alpha (Ero1l) expression in transplanted hearts, accompanied by high levels of NETs (Figure 2J–L). Due to the short lifespan of isolated primary neutrophils, we used the HL‐60 cell line, which has been induced to differentiate and mature, for our molecular mechanism studies. We successfully generated HL‐60 cells with low Ero1l expression (Figure S3I, Supporting Information). Through co‐culture with H/R supernatants, we found that HL‐60 cells with low Ero1l expression exhibited lower NETs generation capacity compared to HL‐60 cells with normal Ero1l expression (Figure 2M,N), indicating that Ero1l is crucial for NETs formation. In conclusion, we identified a subpopulation of neutrophils, Ero1l^+^ neutrophils, in cardiac tissues that is closely associated with NETs induced by myocardial IR injury after HT.

### 2.3 NETs Formation of Ero1l^+^ Neutrophils During Myocardial IR Injury After HT is Closely Associated with M1 Macrophages

**Figure 3:**
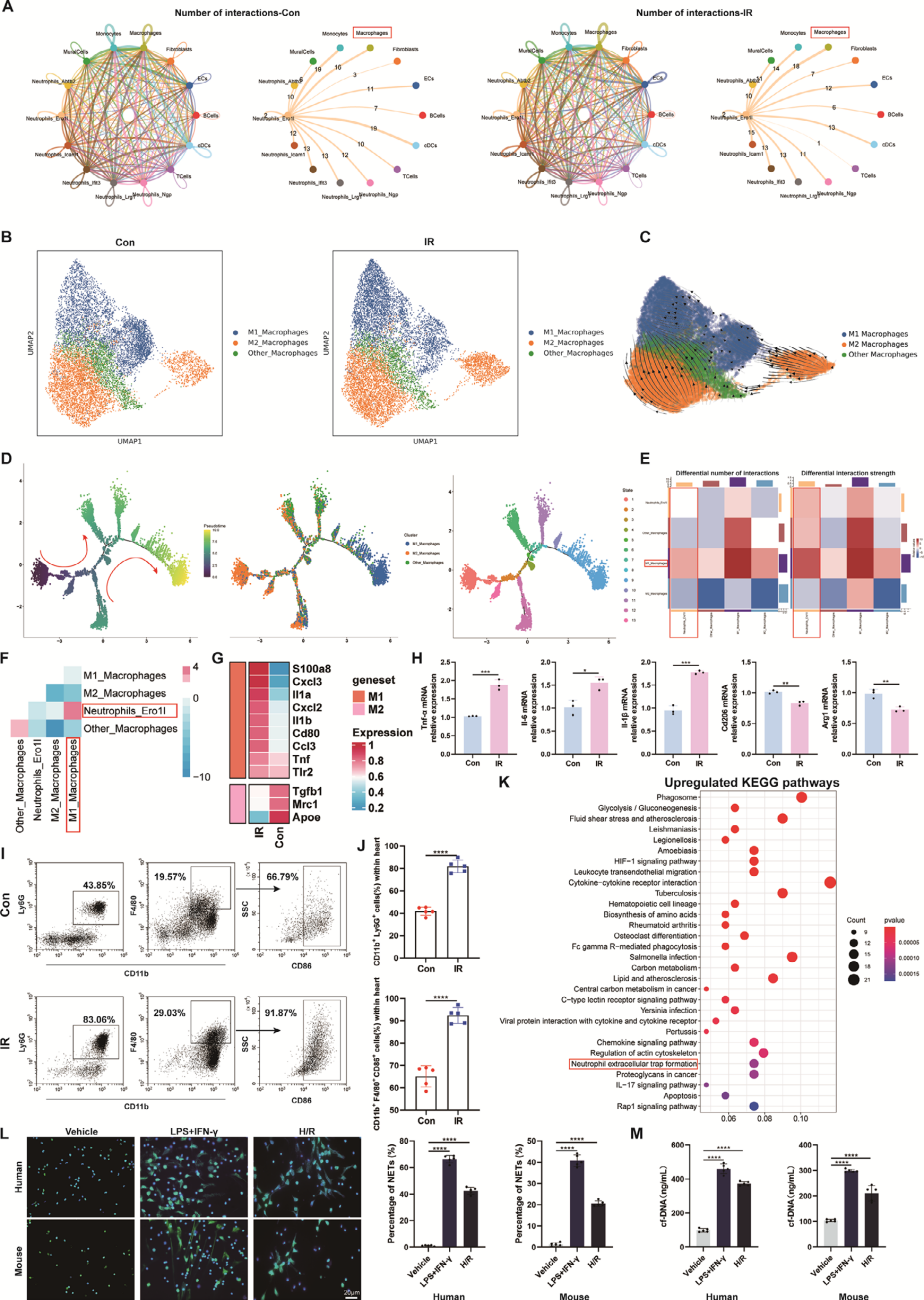
NETosis is strongly associated with M1 macrophages induced by IR after HT. A) Cell‐cell interaction networks and chord diagram showing the potential interaction magnitude between Ero1l^+^ neutrophil subpopulation and other different cell types in each group. The line thickness is proportional to the number of ligand‐receptor pairs. B) UMAP plot of macrophage sub‐clusters in each group. C) RNA velocity of macrophage sub‐clusters. D) The left pseudotime trajectory is shown colored in a gradient from dark blue to yellow, and the start of pseudotime is indicated. The middle pseudotime trajectory is shown colored by different macrophage sub‐clusters. The right pseudotime trajectory is shown colored by different cell states. E) The left panel shows the difference in the number of reciprocal pairs between the different cell types between the two sets, with the receptor cell type in the horizontal coordinates and the ligand cell type in the vertical coordinates, the colored bars at the top represent the sum of the column values displayed in the incoming signals, and the colored bars on the right represent the sum of the row values of the outgoing signals, and in the color columns, the red (or blue) color indicates that the signals in the second dataset increase (or decrease). F) Heatmap of the number of differential interaction pairs between two groups, with red representing more interaction pairs in the IR group and blue representing more interaction pairs in the Con group. G) Heatmap showing differential genes expression in M1 macrophage subpopulation associated with markers of M1 polarization and M2 polarization. H) Gene expression profiles of M1 macrophage markers and M2 macrophage markers in hearts following myocardial IR after HT (*n* = 3). I) Representative flow cytometry plots showing the percentages of neutrophils (CD11b^+^ Ly6G^+^) and M1 macrophages (CD11b^+^ F4/80^+^ CD86^+^) within the heart tissues of control and IR mice. J) Quantification of neutrophils and M1 macrophages within the heart tissues of control and IR mice (*n* = 5). K) KEGG pathway enrichment analysis of up‐regulated differential genes in M1 macrophage subpopulation, which was significantly enriched in the NETs pathway. L) Images and quantitative analysis of NETs formation in human or mouse peripheral blood neutrophils by SYTOX Green staining (*n* = 5). Scale bar = 20 µm. M) The levels of cfDNA in the supernatants of peripheral blood neutrophils co‐cultured with BMDMs induced by LPS/IFN‐γ and H9c2‐derived H/R supernatants (*n* = 5). ns: No significant, ^*^ *p* <0.05, ^**^ *p* <0.01, ^***^ *p* <0.001, ^****^ *p* <0.0001.

Dissecting the interaction between Ero1l^+^ neutrophils and other 8 cell populations in the HT microenvironment will facilitate further elucidation of the mechanism of NETs formation in Ero1l^+^ neutrophils. We found that macrophages had the most active intercellular crosstalk with Ero1l^+^ neutrophils compared to other cells (**Figure** **3**A; Figure S3J, Supporting Information). To further investigate the potential mechanism by which macrophages induce the formation of NETs from Ero1l^+^ neutrophils, we explored the heterogeneity and functions of macrophages within the HT microenvironment. It has been recognized that macrophage‐mediated inflammation plays a central role in myocardial IR injury and repair.^[^ ^33^ ^]^ Macrophages are traditionally classified into M1 and M2 macrophages, roughly corresponding to pro‐inflammatory and anti‐inflammatory phenotypes, respectively. Therefore, we performed a centralized clustering analysis of macrophages and further separated them into 3 main clusters, including M1 macrophages, M2 macrophages, and other types of macrophages (Figure 3B; Figure S4A, Supporting Information). The percentage of M1 macrophages increased after HT, and trajectory analysis clearly demonstrated that M1 macrophages were differentiated from the other 2 macrophage subsets (Figure 3C,D; Figure S4B, Supporting Information). CellChat was utilized to predict putative interaction between Ero1l^+^ neutrophils and macrophage clusters. We observed a significant interaction between Ero1l^+^ neutrophils and M1 macrophages (Figure 3E). Complementary analysis using CellPhoneDB, which assesses cell‐cell interactions based on ligand‐receptor pairs, also identified M1 macrophages as the cell population most densely connected to Ero1l⁺ neutrophils (Figure 3F). In addition, the high expression of M1‐related genes and low expression of M2‐related genes in the M1 macrophage subpopulation further fully confirmed the M1‐polarized character of this subpopulation (Figure 3G; Table S2, Supporting Information). Taken together, given the active participation of M1 macrophages in intercellular interactions, we suggested that M1 macrophage polarization plays an important role in the NETosis pathogenesis of IR injury in HT.

Notably, we found that the M2 macrophage subpopulation exhibited two separate and distinct cell populations. Given the complex heterogeneity of macrophages in transplanted hearts, we applied another dimensionality reduction analysis for macrophage subpopulations (Figure S4C, Supporting Information). Remarkably, the M1 macrophage cluster was divided into Cxcl3^+^ macrophages and Peak1^+^ macrophages, while the M2 macrophage cluster consisted of two macrophage subpopulations, Ccl5^+^ macrophages and Fabp5^+^ macrophages (Figure S4D, Supporting Information). Interestingly, the two M2 macrophage subpopulations exhibited similar gene expression profiles and biological pathways (Figure S4E–G, Supporting Information). Considering that M1 macrophages were the subpopulation of interest in this study, our subsequent analysis still focused on the M2 macrophage subpopulation as a whole.

Subsequently, we asked whether cardiac transplant reperfusion injury could polarize macrophages toward the M1 phenotype. Based on single‐cell transcriptomics data, classical M1 marker genes (Tnf‐α and Il‐1β) were significantly upregulated in cardiac total macrophages after HT (Figure S5A, Supporting Information). The reverse transcription‐quantitative polymerase chain reaction (RT‐qPCR) analysis for M1 and M2 gene expression in myocardial tissues also showed that M1 markers (Tnf‐α, Il‐6, and Il‐1β) were significantly increased, whereas the M2 markers were reduced, such as Arg1 and Cd206 (Figure 3H). Interestingly, flow cytometry analysis also indicated that compared with the control group, the proportion of M1 macrophages (CD11b^+^ F4/80^+^ CD86^+^) and neutrophils (CD11b^+^ Ly6G^+^) was remarkably increased in myocardial tissues of the IR group (Figure 3I,J; Figure S5B, Supporting Information). Western blotting showed that macrophages co‐incubated with H/R supernatants were polarized toward the M1 phenotype but not the M2 phenotype (Figure S5C,D, Supporting Information). Collectively, these data demonstrate that macrophages could polarize from the M2 phenotype to an M1‐like state under myocardial IR injury in HT.

Notably, KEGG pathway analysis showed that genes upregulated in M1 macrophages following IR injury were enriched in the NETs pathway (Figure 3K). Subsequently, to further validate the direct effect of myocardial IR injury‐induced M1 macrophages on neutrophils, we induced bone marrow‐derived macrophages (BMDMs) using cardiomyocyte‐derived H/R supernatants and then co‐cultured them with human peripheral blood neutrophils by a co‐culture system (Figure S6A, Supporting Information). As a positive control, M1‐BMDMs were induced using lipopolysaccharide (LPS) and interferon‐gamma (IFN‐γ). Interestingly, M1‐BMDMs generated using H/R supernatants promoted NETosis to a comparable extent as the LPS/IFN‐γ‐induced positive controls (Figure 3L,M). Taken together, these results support the idea that M1‐type macrophages induced by myocardial IR injury after HT participate in the formation of NETs in Ero1l^+^ neutrophils.

### 2.4 M1 Macrophages Induce NETosis via the THBS1/CD47 Axis

**Figure 4:**
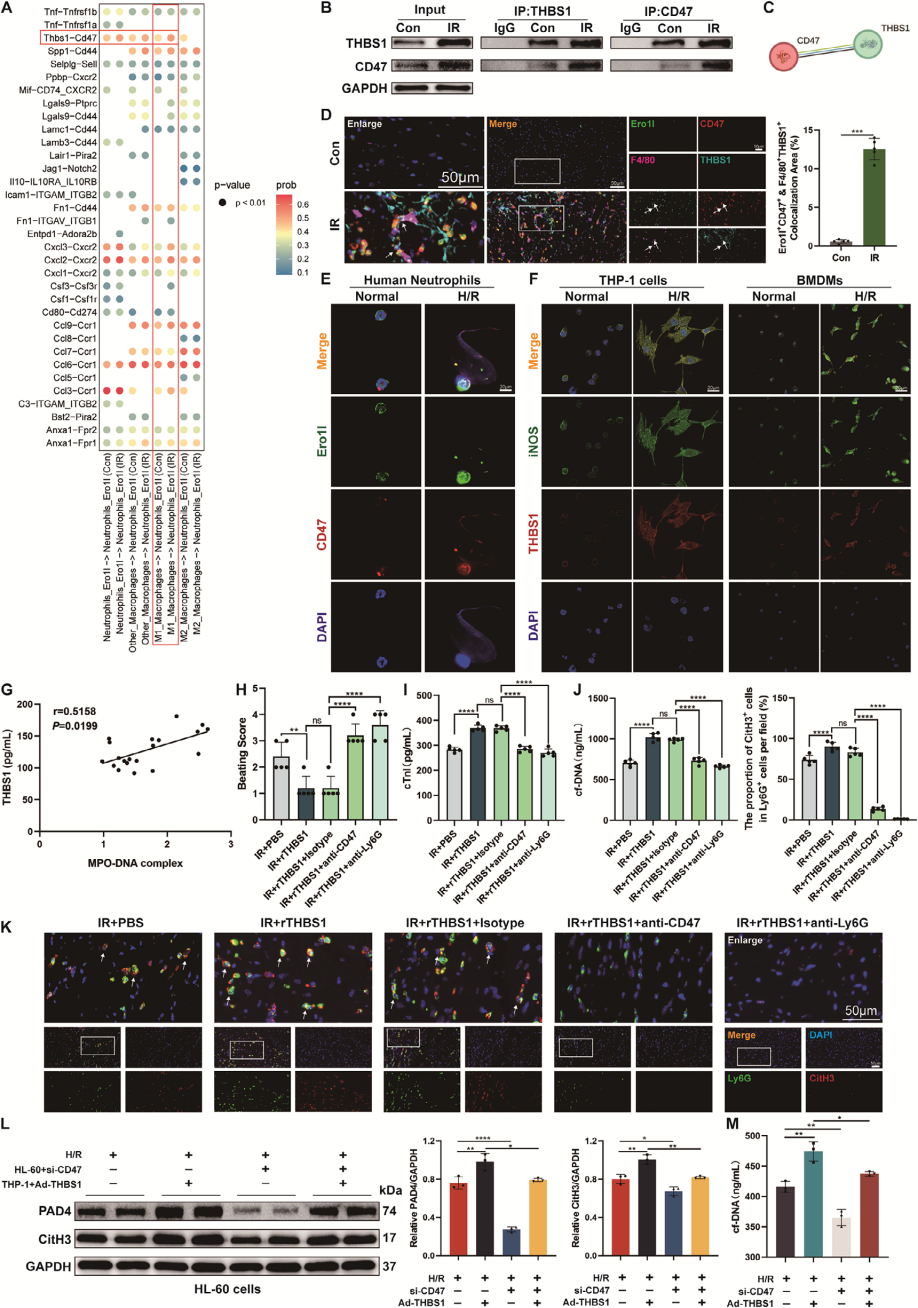
M1 macrophages could regulate IR‐mediated NETosis and myocardial injury in HT through the THBS1/CD47 pathway. A) Dot plot of selected ligand‐receptor interaction between Ero1l^+^ neutrophils and macrophage subpopulations in Con and IR hearts. The size of the dots represents the interactions' significant values and the color of the dots represents the intensity of interactions. B) Co‐immunoprecipitation assay to detect the interaction between THBS1 and CD47 in myocardial tissues. C) The PPI network is constructed based on the STRING database. D) Immunofluorescence co‐staining for Ero1l (green), CD47 (red), F4/80 (magenta), and THBS1 (cyan) in the hearts of mice. Scale bar = 50 µm. E) Immunofluorescence co‐staining for Ero1l (green) and CD47 (red) in human peripheral blood neutrophils co‐cultured with BMDMs induced by H9c2‐derived normal and H/R supernatants. Scale bar = 20 µm. F) Immunofluorescence co‐staining for iNOS (green) and THBS1 (red) in BMDMs and THP‐1 cells co‐cultured with H9c2‐derived normal and H/R supernatants. Scale bar = 20 µm. G) The Spearman correlation analysis of THBS1 and MPO‐DNA complex in serum (*n* = 20). H) Beating score measured by the Stanford Cardiac Surgery Laboratory graft scoring system (*n* = 5). I) The serum levels of cTnI were evaluated in different groups of mice (*n* = 5). J) The levels of cfDNA in peripheral blood (*n* = 5). K) Representative immunofluorescence images and quantification of Ly6G^+^ CitH3^+^ double‐positive cells with staining for Ly6G (green) and CitH3 (red) in heart tissues (*n* = 5). Scale bar = 50 µm. L) The levels of PAD4 and CitH3 proteins in HL‐60 cells co‐cultured with THP‐1 cells induced by H9c2‐derived H/R supernatants were measured by western blotting (*n* = 3). M) The levels of cfDNA in the supernatants of HL‐60 cells co‐cultured with THP‐1 cells induced by H9c2‐derived H/R supernatants (*n* = 3). ns: No significant, ^*^ *p* <0.05, ^**^ *p* <0.01, ^***^ *p* <0.001, ^****^ *p* <0.0001.

To elucidate the molecular processes by which M1 macrophages regulate the phenotype of Ero1l^+^ neutrophils at the single‐cell level, we analyzed macrophage‐driven ligand‐receptor pairs. As shown in the dot plots, there were differentially regulated signals between control and IR group hearts (**Figure** **4**A), where differences in THBS1‐CD47, a ligand‐receptor pair, were evident between the M1 macrophage subpopulation and Ero1l^+^ neutrophils. The violin plots showed that, compared with the Con group, the expression levels of Thbs1 in the M1 macrophage subpopulation and Cd47 in the Ero1l^+^ neutrophil subpopulation were significantly increased after IR (Figure S6B,C, Supporting Information). Importantly, Thbs1 levels in M1 macrophages after HT were significantly higher than in the other two macrophage subpopulations, while Cd47 levels were also relatively high in Ero1l^+^ neutrophils among 6 neutrophil subpopulations (Figure S6D,E, Supporting Information). We performed a co‐immunoprecipitation assay to validate the predicted interaction, and the results showed that the interaction between THBS1 and CD47 increased significantly in the IR group compared to the control group (Figure 4B). The interaction network of THBS1 and CD47 was further confirmed using the STRING database (Figure 4C). Moreover, immunofluorescence experiments showed obvious co‐localization of F4/80, THBS1, Ero1l, and CD47 in transplanted hearts (Figure 4D). In the myocardial tissues of the IR group, the proportion of CD47^+^ cells among Ero1l^+^ neutrophils was significantly increased, as was the proportion of THBS1^+^ cells among M1 macrophages (Figure S6F,G, Supporting Information). To further explore the cell specificity of co‐localization, we performed immunofluorescence on neutrophils and macrophages. Consistent with the above results, we observed that the formation of NETs by neutrophils was accompanied by increased expression of CD47 and Ero1l after the addition of H/R supernatants to the co‐culture system of neutrophils and macrophages (Figure 4E). Notably, immunofluorescence staining also demonstrated that THBS1 and iNOS were overexpressed simultaneously in BMDMs and THP‐1 cells (Figure 4F). Furthermore, to determine the level of external secretion of THBS1 co‐cultured with H/R supernatants, we ultrafiltrated the cellular supernatants and performed enzyme‐linked immunosorbent assay (ELISA) analysis. Our data showed that the secretion of the THBS1 protein was increased in THP‐1 cells and BMDMs induced by H/R supernatants, indicating that M1 macrophages are one of the sources of THBS1 secretion after HT (Figure S6H, Supporting Information).

Clinical data showed that THBS1 levels in the serum of heart transplant patients were significantly positively correlated with NETs levels (Figure 4G). To explore the role of the THBS1/CD47 axis in myocardial IR injury and NETosis after HT, we administered anti‐THBS1 or control IgG to HT recipient mice. Anti‐THBS1 did not alter cardiac beating scores but significantly attenuated myocardial injury (Figure S7A–C, Supporting Information). And we observed a reduction in NETs levels of neutrophils after anti‐THBS1 treatment (Figure S7D, Supporting Information). In addition, hematogenous macrophages and circulating monocytes were depleted with clodronate liposomes (Clo‐Lip) before the recipient mice suffered HT. We found that compared with the IR group, myocardial injury was significantly attenuated and the level of NETs was significantly reduced after depletion of macrophages (Figure S7A–D, Supporting Information). These results were not significantly different from those of the IR + anti‐THBS1 group, suggesting that most of the THBS1 that induced NETosis and myocardial injury in transplanted hearts originated from monocyte‐derived macrophages recruited from the recipients. Furthermore, recombinant THBS1 (rTHBS1) was injected into transplanted hearts as an exogenous supplement to investigate the role of THBS1 in myocardial IR injury after HT. Compared to PBS, IR injury was even worse with rTHBS1 treatment (Figure 4H,I). Additionally, the NETs levels were significantly increased in the hearts of rTHBS1‐treated mice (Figure 4J,K). To demonstrate that rTHBS1 exerts these effects by acting on neutrophils in donor hearts, anti‐Ly6G neutralizing antibody was used to deplete circulating neutrophils in recipient mice. Neutrophil depletion remarkably reversed cardiac IR injury exacerbated by rTHBS1 and improved grafted cardiac function while reducing myocardial NETs levels (Figure 4H–K). Moreover, we administered anti‐CD47 to deplete CD47 in mice, which similarly attenuated myocardial IR injury and NETs production induced by rTHBS1 after HT (Figure 4H–K). These results suggest that macrophages recruited from recipient mice could induce neutrophil CD47 activation and NETs phenotype by producing THBS1 mediators.

Subsequently, we further validated our findings through in vitro experiments. We successfully generated THBS1‐overexpressing THP‐1 cells (Figure S8A, Supporting Information), which were co‐cultured with HL‐60 cells after induced differentiation in cardiomyocyte‐derived H/R supernatants. HL‐60 cells were treated with si‐CD47 or control siRNA for silencing CD47. The knockdown efficiency of CD47 by siRNA was evaluated via western blotting (Figure S8B, Supporting Information). Interestingly, knockdown of CD47 in neutrophils inhibited the promotion of NETosis by THBS1 overexpressing THP‐1 cells (Figure 4L,M). Taken together, all these results illustrate that the THBS1/CD47 axis activated by M1 macrophages recruited from recipient mice might play a critical role in promoting NETosis and exacerbating myocardial IR injury after HT.

### 2.5 CD47 Promotes NETs Formation Via Activating p38 MAPK Signaling Pathway

**Figure 5:**
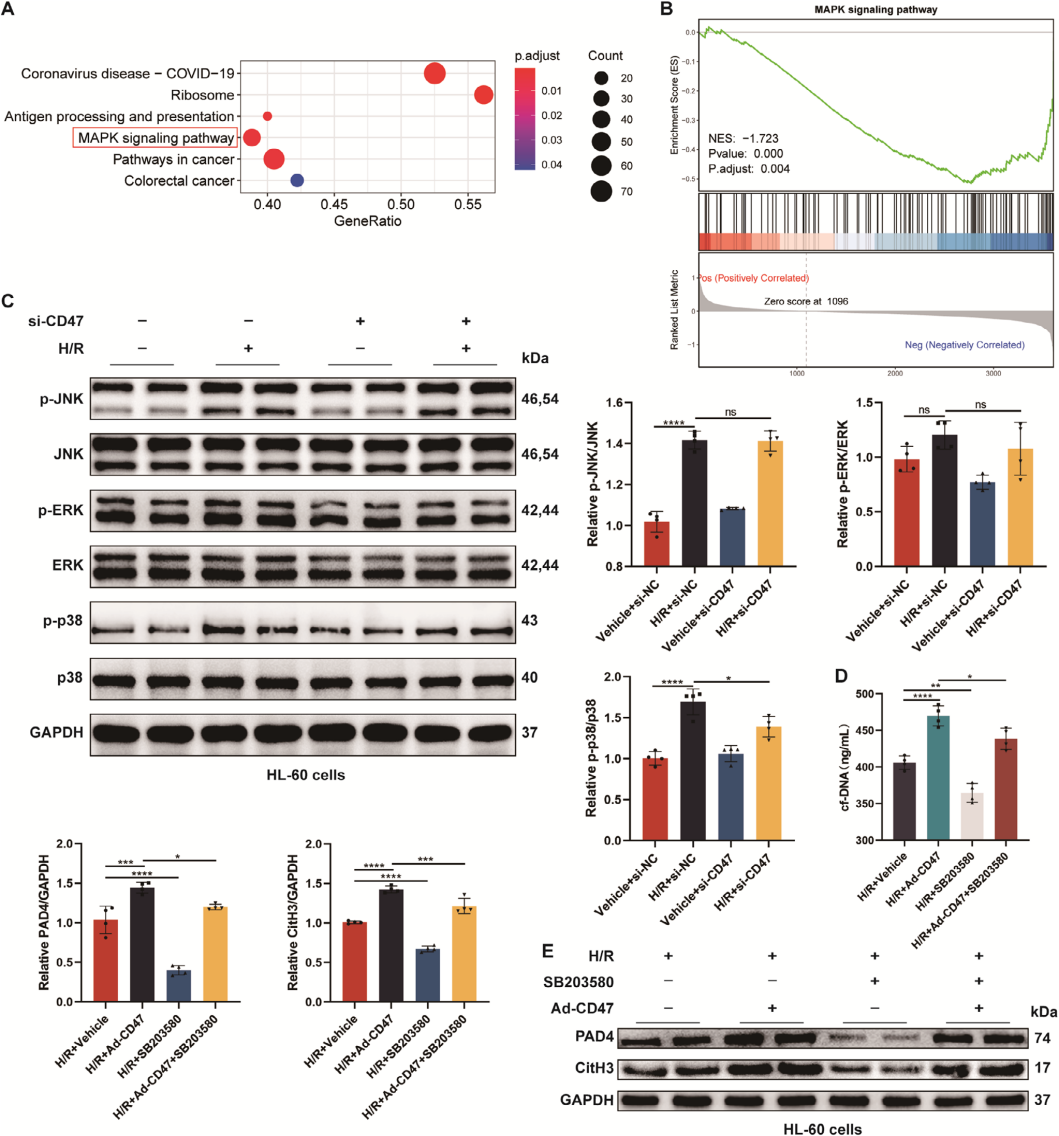
CD47 mediates NETosis through the p38 MAPK signaling pathway. A, B) GSEA showed MAPK signaling pathway was activated in Ero1l^+^ neutrophils after HT. C) The levels of MAPK signaling pathway proteins in HL‐60 cells co‐cultured with THP‐1 cells induced by H9c2‐derived normal and H/R supernatants were measured by western blotting (*n* = 4). D) The levels of cfDNA in the supernatants of HL‐60 cells co‐cultured with THP‐1 cells induced by H9c2‐derived H/R supernatants (*n* = 4). E) The levels of PAD4 and CitH3 proteins in HL‐60 cells co‐cultured with THP‐1 cells induced by H9c2‐derived H/R supernatants were measured by western blotting (*n* = 4). ns: No significant, ^*^ *p* <0.05, ^**^ *p* <0.01, ^***^ *p* <0.001, ^****^ *p* <0.0001.

Next, to further elucidate the intrinsic molecular mechanism of the THBS1/CD47 axis‐induced NETosis during cardiac transplant reperfusion, gene set enrichment analysis (GSEA) was performed. We observed that the enrichment of mitogen‐activated protein kinases (MAPK) signaling pathway in the Ero1l^+^ neutrophils after HT (**Figure** **5**A,B). Some studies have shown that activation of the MAPK pathway could induce NETs generation.^[^ ^34^ ^]^ By performing immunoblotting, we observed that both c‐Jun N‐terminal kinase (JNK) and p38 enzymes were activated after co‐cultured with THP‐1 cells induced by H9c2‐derived H/R supernatants (Figure 5C). Next, we assessed the effect of CD47 on MAPK pathway activation in HL‐60 cells co‐cultured with THP‐1 cells induced by H9c2‐derived H/R supernatants. The results showed that CD47 deficiency significantly reversed the level of p‐p38, but had little effect on p‐JNK expression (Figure 5C). In addition, we generated HL‐60 cells with overexpressed CD47 (Figure S8C, Supporting Information), and found that CD47 knockout or blocking of THBS1/CD47 could significantly reduce NETs generation (Figure 4L,M), whereas CD47 overexpression presented the opposite effect (Figure 5D,E). Importantly, pharmacological inhibition of p38 MAPK using SB203580, a specific p38 inhibitor, significantly suppressed NETosis driven by ad‐CD47 (Figure 5D,E). These findings support the fact that CD47 promotes NETosis by modulating activation of the p38 MAPK signaling pathway during myocardial IR.

### 2.6 ARID3A Regulates THBS1 Expression in M1 Macrophages at the Transcriptional Level

**Figure 6:**
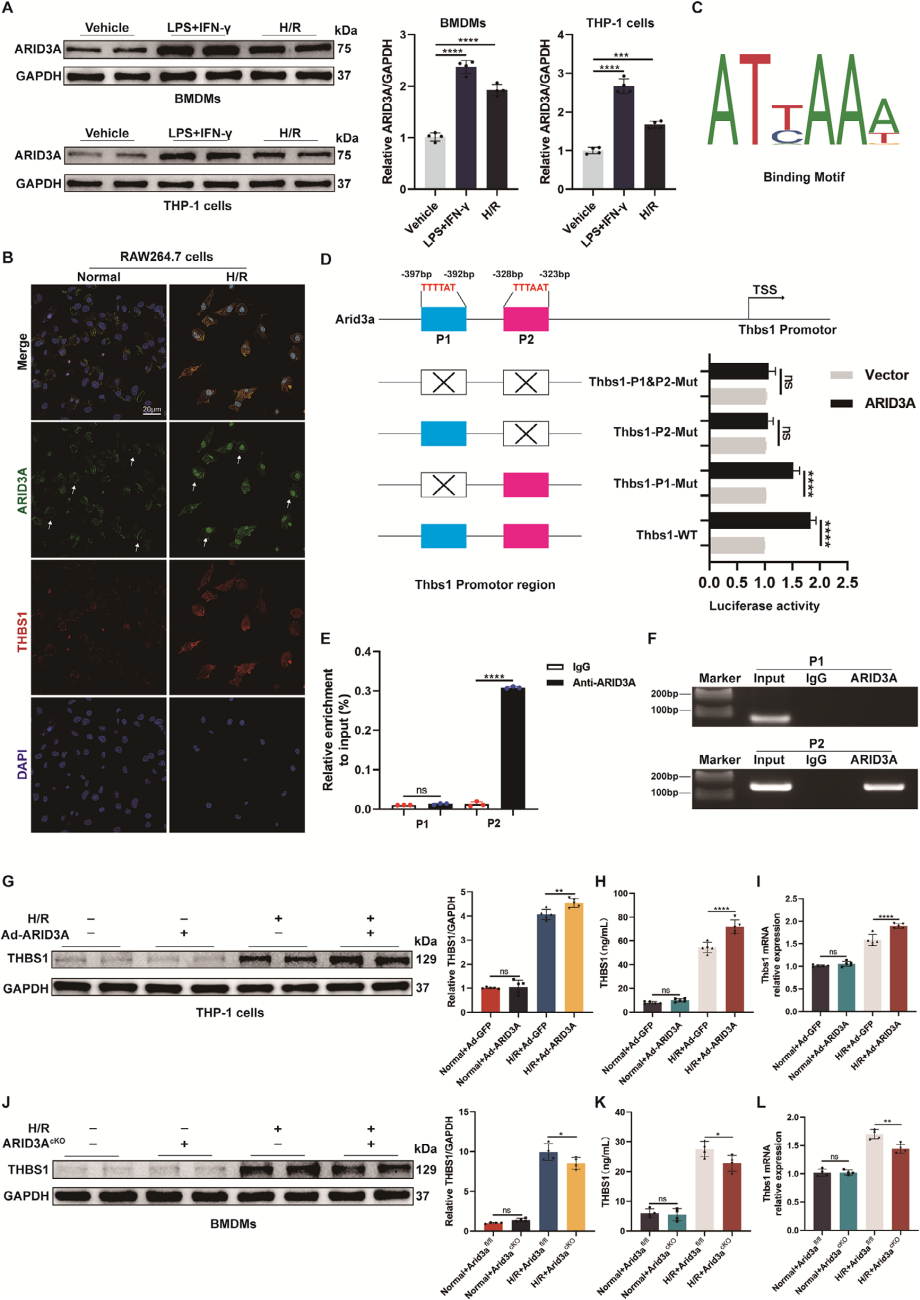
The ARID3A of macrophages could regulate THBS1 at the transcriptional level. A) The levels of ARID3A protein in BMDMs and THP‐1 cells induced by LPS/IFN‐γ and H9c2‐derived H/R supernatants were measured by western blotting (*n* = 4). B) Immunofluorescence co‐staining for ARID3A (green) and THBS1 (red) in RAW264.7 cells co‐cultured with H9c2‐derived normal and H/R supernatants. Scale bar = 20 µm. C) Schematic diagram of ARID3A binding motif predicted based on the JASPAR database. D) Dual‐luciferase reporter assay to detect the THBS1 promoter activity. E) ChIP assay was performed to analyze ARID3A binding to the THBS1 promoter using an anti‐ARID3A antibody. F) Agarose gel electrophoresis for ChIP assay to validate ARID3A binding to the promoter region of the Thbs1 gene. G, I) The levels of THBS1 protein and mRNA in THP‐1 cells co‐cultured with H9c2‐derived normal or H/R supernatants (*n* = 5). H, K) Quantification of THBS1 secreted by THP‐1 cells and BMDMs. J, L) The levels of THBS1 protein and mRNA in ARID3A^cKO^ mice‐derived BMDMs co‐cultured with H9c2‐derived normal or H/R supernatants (*n* = 4). ns: No significant, ^*^ *p* <0.05, ^**^ *p* <0.01, ^***^ *p* <0.001, ^****^ *p* <0.0001.

To further explore the mechanisms by which THBS1 is modulated in M1 macrophages, we employed pySCENIC to identify transcription factors specifically activated in a subpopulation of M1 macrophages at the single‐cell level. Several transcription factors were upregulated in M1 macrophages, such as ARID3A, ARG2, KLF7, ZFP825 and STAT6 (Figure S9A, Supporting Information). In addition, ENCODE (https://www.encodeproject.org/) and hTFtarget (https://guolab.wchscu.cn/hTFtarget/#! /) were used as publicly available databases to predict transcription factors that may be involved in THBS1 regulation through interaction with its promoter region (Table S3, Supporting Information). By intersecting them with the results of our pySCENIC analysis, we finally identified ARID3A as a significantly upregulated transcription factor in M1 macrophages (Figure S9B,C, Supporting Information). Consistent with this prediction, ARID3A protein expression was consistently elevated in macrophages co‐cultured with LPS and IFNγ and in macrophages co‐cultured with H/R supernatants (**Figure** **6**A). Immunofluorescence staining showed a significant enhancement of the fluorescence intensity of ARID3A and THBS1 in RAW264.7 cells treated with H/R supernatants, while significant nuclear translocation of ARID3A was observed (Figure 6B).

To determine whether ARID3A directly regulates THBS1 transcription, we conducted dual‐luciferase reporter assays and chromatin immunoprecipitation (ChIP) analysis. According to the prediction of the database JASPAR (http://jaspar.genereg.net/), we selected two potential ARID3A binding sites in the THBS1 promoter (Figure 6C). Subsequently, we constructed wild‐type (WT) and mutant type (Mut) THBS1 promoter‐luciferase reporter plasmids and performed dual‐luciferase reporter gene assays. As shown in Figure 6D, ARID3A overexpression significantly elevated luciferase activity in cells after co‐transfection with WT THBS1 and Mut P1‐THBS1. Nevertheless, luciferase activity remained unchanged when co‐transfected with Mut P2‐THBS1 plasmid and Mut P1&P2‐THBS1 plasmid. To validate direct binding of ARID3A to the P2 region, we performed ChIP‐qPCR, which demonstrated strong enrichment of ARID3A at the P2 site. This finding was further confirmed by 3% agarose gel electrophoresis (Figure 6E,F). Together, these results collectively confirm that ARID3A directly binds to the P2 region of the THBS1 promoter and drives its transcription.

To explore the role of ARID3A on THBS1 in H/R injury‐induced M1 macrophages, we overexpressed ARID3A in THP‐1 cells using adenoviral vectors (Figure S9D, Supporting Information). ARID3A‐overexpressing macrophages exhibited significantly increased THBS1 secretion and expression in response to H/R supernatants, as evidenced by elevated protein levels in cell culture supernatants and increased mRNA and intracellular protein expression in THP‐1 cells (Figure 6G–I). To further confirm these results, we constructed myeloid‐specific ARID3A knockout mice (Figure S9E, Supporting Information), and the primary macrophages, BMDMs, were generated from ARID3A^fl/fl^ and ARID3A^cKO^ mice. The genotype of the mice was verified by agarose gel electrophoresis, and the results showed remarkably decreased levels of ARID3A expression in BMDMs derived from bone marrow of ARID3A^cKO^ mice (Figure S9F–H, Supporting Information). Accordingly, when BMDMs extracted from ARID3A^fl/fl^ and ARID3A^cKO^ mice were treated with H/R supernatants, we found that BMDMs from ARID3A^cKO^ mice exhibited lower levels of THBS1 expression and secretion (Figure 6J–L). Taken together, our findings suggest that the transcription factor ARID3A drives the transcription of THBS1 in M1 macrophages induced by myocardial IR injury after HT.

### 2.7 ARID3A Regulates NETs Formation and Myocardial IR Injury After HT Through the THBS1/CD47 Signaling Pathway

**Figure 7:**
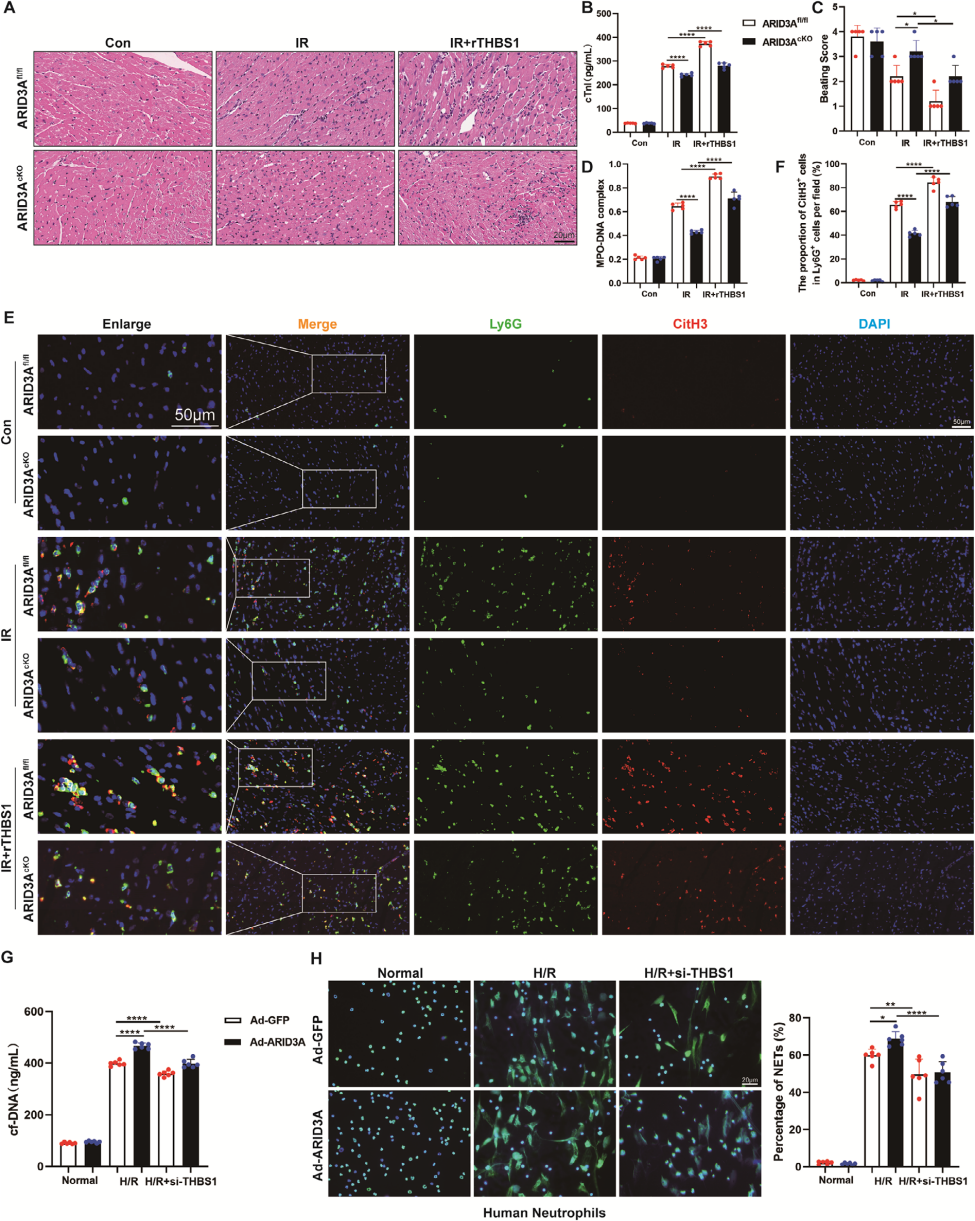
Macrophage ARID3A deficiency ameliorates IR injury in HT by modulating THBS1/CD47‐mediated NETosis. A) Representative image of H&E staining of heart tissues. Scale bar = 20 µm. B) The serum levels of cTnI were evaluated in different groups of mice (*n* = 5). C) Beating score measured by the Stanford Cardiac Surgery Laboratory graft scoring system (*n* = 5). D) Quantitative analysis of MPO‐DNA complex in serum of mice (*n* = 5). E, F) Representative immunofluorescence images and quantification of Ly6G^+^ CitH3^+^ double‐positive cells with staining for Ly6G (green) and CitH3 (red) in heart tissues (*n* = 5). Scale bar = 50 µm. G) The levels of cfDNA in the supernatants of human peripheral blood neutrophils co‐cultured with THP‐1 cells (*n* = 6). H) Images and quantitative analysis of NETs formation in human peripheral blood neutrophils co‐cultured with THP‐1 cells by SYTOX Green staining (*n* = 6). Scale bar = 20 µm. ns: No significant, ^*^ *p* <0.05, ^**^ *p* <0.01, ^***^ *p* <0.001, ^****^ *p* <0.0001.

To explore the role of macrophage ARID3A in myocardial IR injury after HT, we performed HT in ARID3A^cKO^ mice. We observed a significant reduction in neutrophil infiltration, attenuation of myocardial injury, and a significant increase in beating scores in transplanted hearts of ARID3A^cKO^ mice compared with ARID3A^fl/fl^ mice (**Figure** **7**A–C). Accordingly, the mRNA levels of pro‐inflammatory cytokines Tnf‐α and Il‐6 were markedly decreased in the ARID3A^cKO^ group after myocardial IR compared with the ARID3A^fl/fl^ group. There were no differences in Arg1 and Cd206 mRNA levels in ARID3A^cKO^ mice compared with ARID3A^fl/fl^ mice under normal conditions, whereas myeloid‐specific ARID3A knockout significantly upregulated these markers after myocardial IR injury (Figure S10A, Supporting Information). In addition, ARID3A^cKO^ mice displayed significantly lower circulating levels of MPO‐DNA complex (Figure 7D). Immunofluorescence staining showed reduced co‐localization of Ly6G and CitH3 in the myocardium of ARID3A^cKO^ mice after HT, confirming the lower levels of NETs in myocardial tissues (Figure 7E,F). To further examine the involvement of ARID3A in THBS1‐mediated NETosis and myocardial IR injury, we administered rTHBS1 to both ARID3A^fl/fl^ and ARID3A^cKO^ mice after HT. Notably, THBS1 supplementation abolished the protective effects observed in ARID3A^cKO^ mice, as evidenced by disorganized myocardial fiber alignment, enhanced neutrophil infiltration, increased serum cTnI levels, and reduced beating scores (Figure 7A–C). The decreased NETs levels in transplanted hearts and circulation were also reversed after administration of rTHBS1 to ARID3A^cKO^ mice (Figure 7D–F).

To validate these findings in vitro, we transfected THP‐1 cells with siRNA targeting THBS1. Successful knockdown was confirmed by western blotting analysis (Figure S10B, Supporting Information). Our results showed that NETs formation was significantly enhanced when THP‐1 cells overexpressing ARID3A were stimulated with H/R supernatants and co‐cultured with human peripheral blood neutrophils. Yet, in the H/R group when ARID3A was overexpressed and THBS1 was silenced at the same time, the effect of THP‐1 cells overexpressing ARID3A on NETs generation was weakened (Figure 7G,H). These data demonstrated that si‐THBS1 abolishes the enhanced effect of ARID3A on NETosis. In conclusion, we demonstrated that ARID3A could modulate NETosis and transplanted cardiac IR injury by affecting the THBS1/CD47 axis.

### 2.8 4‐OI Ameliorates Myocardial IR Injury by Suppressing M1 Macrophage Polarization and NETosis

**Figure 8:**
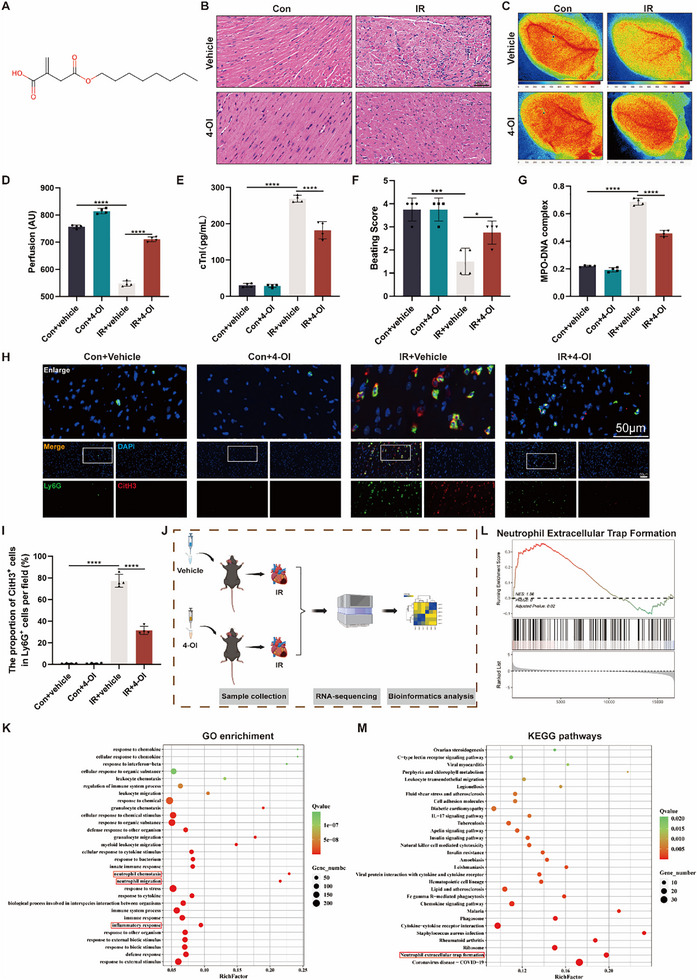
Exogenous 4‐OI administration ameliorates IR injury and inhibits NETosis during HT. A) The chemical structure of 4‐OI. B) Representative image of H&E staining of heart tissues. Scale bar = 20 µm. C, D) Representative peak perfusion images in AU and statistical analysis of mean perfusion (*n* = 4). Pseudo‐colored scale bar represents level of perfusion with higher values in red and lower values in blue. E) The serum levels of cTnI were evaluated in different groups of mice (*n* = 4). F) Beating score measured by the Stanford Cardiac Surgery Laboratory graft scoring system (*n* = 4). G) ELISA analysis for MPO‐DNA complex levels in serum (*n* = 4). H, I) Representative immunofluorescence images and quantification of Ly6G^+^ CitH3^+^ double‐positive cells with staining for Ly6G (green) and CitH3 (red) in heart tissues (*n* = 4). Scale bar = 50 µm. J) The schematic of the RNA‐seq between IR mice and IR + 4‐OI mice (*n* = 3). K) Results of DEGs for GO enrichment research (Biological Process). L) GSEA showed that the NETs pathway in the IR + 4‐OI group is enriched. M) KEGG pathway enrichment analysis showed that DEGs are enriched in the NETs pathway. ns: No significant, ^*^ *p* <0.05, ^**^ *p* <0.01, ^***^ *p* <0.001, ^****^ *p* <0.0001.

Although the regulatory mechanisms of NETosis in myocardial IR injury after HT have been preliminarily elucidated, therapeutic strategies remain limited. Given this, we next aimed to target M1 macrophage polarization as a potential intervention point for ameliorating myocardial IR injury after HT. The metabolite itaconate and itaconate derivatives have demonstrated excellent properties that suppress inflammatory responses in macrophages.^[^ ^35^ ^]^ Therefore, we analyzed the role of the 4‐OI in myocardial IR injury during HT. The chemical structure of 4‐OI was presented in **Figure** **8**A. Compared with the IR + vehicle group, administration of 4‐OI significantly attenuated myocardial injury, decreased serum cTnI levels, and increased beating scores and blood flow in the transplanted heart (Figure 8B–F). In addition, we examined the levels of NETs in the heart and circulation of mice. Compared with untreated IR mice, mice in the IR + 4‐OI group had reduced serum levels of MPO‐DNA complexes and decreased co‐localization of Ly6G and CitH3 in the myocardium (Figure 8G–I). Thus, these findings confirm that 4‐OI administration plays a beneficial role in myocardial IR injury and NETosis during HT. To address this hypothesis, we performed RNA Sequencing (RNA‐seq) of transplanted hearts with and without 4‐OI administration (Figure 8J). GO analysis showed that DEGs were significantly enriched in the pathways of neutrophil chemotaxis, neutrophil migration, and inflammatory response (Figure 8K). Moreover, KEGG pathway enrichment and GSEA indicated DEGs were significantly enriched in Neutrophil extracellular trap formation (Figure 8L,M), consistent with our in vivo results. Interestingly, the heatmap of M1‐ and M2‐related gene expression profiles demonstrated a general downregulation of M1 genes and upregulation of M2 genes in the 4‐OI + IR group relative to the IR group (Figure S10C, Supporting Information). Accordingly, we performed immunofluorescence staining using fluorescently labeled F4/80 and CD86, and found that 4‐OI treatment significantly inhibited M1 polarization of macrophages in transplanted hearts (Figure S10D, Supporting Information).

To validate our findings at the cellular level, cardiomyocytes were treated with different concentrations of 4‐OI and then subjected to H/R. CCK‐8 assays indicated that 4‐OI significantly restored the viability of H9c2 and HL‐1 cells, with 100 µm identified as the optimal dose for both cell lines (Figure S11A,B, Supporting Information). Subsequently, we examined the effect of 4‐OI on H/R supernatants induced M1 polarization in macrophages. Flow cytometry analysis showed a significant increase in the proportion of M2 macrophages and a significant decrease in the proportion of M1 macrophages in the H/R + 4‐OI group compared with the H/R group (Figure S11C, Supporting Information). Furthermore, the RT‐qPCR analysis also showed that M1 markers (Tnf‐α, Il‐1β, and Il‐6) were significantly reduced, whereas the M2 markers were increased, such as Arg1 and Cd206 in BMDMs after 4‐OI treatment (Figure S11D, Supporting Information). Overall, the above results suggested that exogenous administration of the itaconate derivative, 4‐OI, which could attenuate myocardial IR injury by inhibiting M1 macrophage polarization and NETosis during HT.

### 2.9 4‐OI could target ARID3A in macrophages to exert therapeutic effects

**Figure 9:**
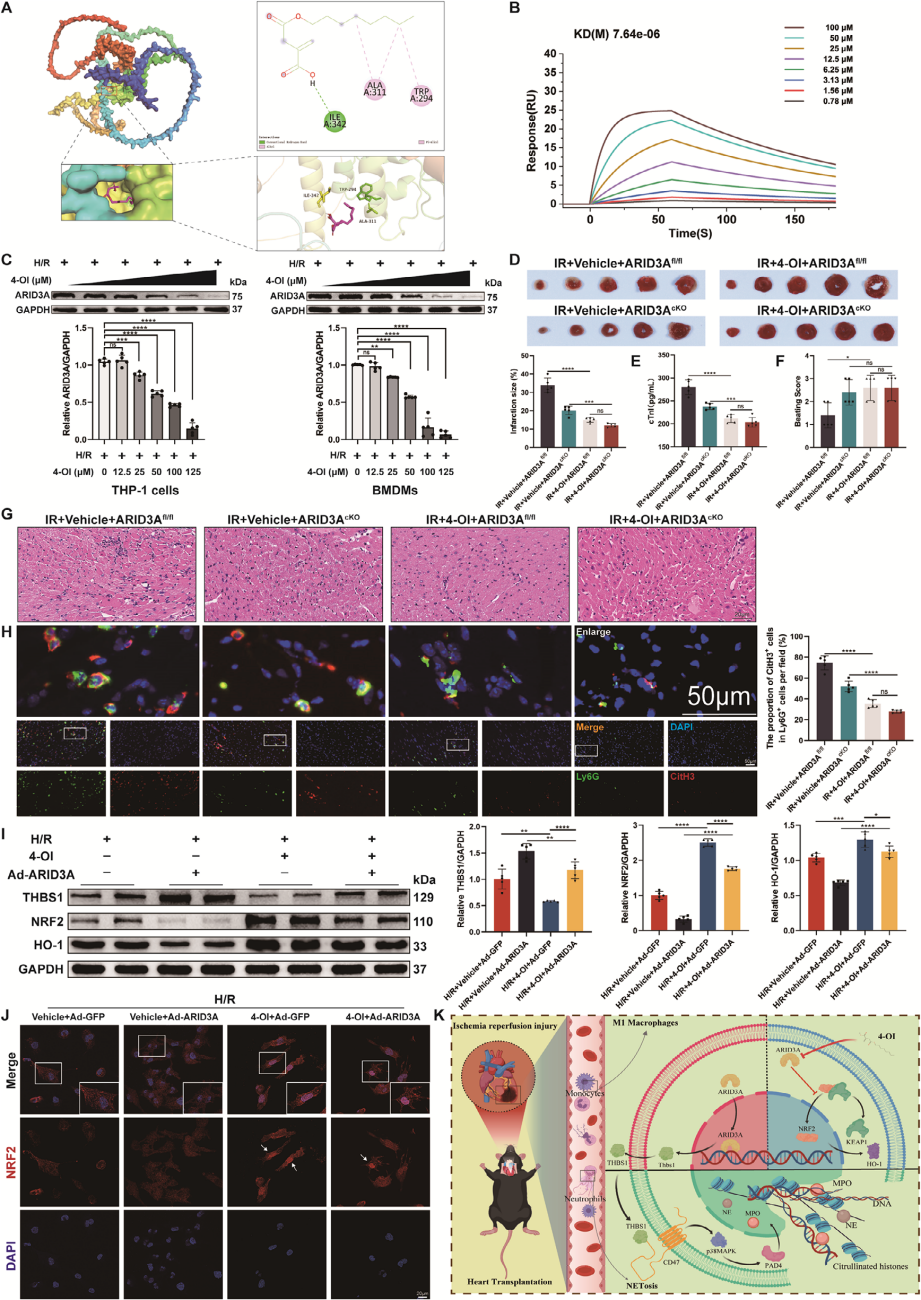
The 4‐OI may target macrophage ARID3A to exert therapeutic effects. A) A molecular docking simulation was performed to investigate the interaction between 4‐OI and ARID3A. B) The SPR assays showing the binding of 4‐OI to ARID3A protein. C) The levels of ARID3A protein in BMDMs and THP‐1 cells induced by 4‐OI and H9c2‐derived H/R supernatants were measured by western blotting (*n* = 5). D) Myocardial infarct size was measured by TTC staining and expressed as the percentage of infarct relative to the total area (*n* = 5). E) The serum levels of cTnI were evaluated in different groups of mice (*n* = 5). F) Beating score measured by the Stanford Cardiac Surgery Laboratory graft scoring system (*n* = 5). G) Representative image of H&E staining of heart tissues. Scale bar = 20 µm. H) Representative immunofluorescence images and quantification of Ly6G^+^ CitH3^+^ double‐positive cells with staining for Ly6G (green) and CitH3 (red) in heart tissues (*n* = 5). Scale bar = 50 µm. I) The levels of NRF2, HO‐1, and THBS1 proteins in THP‐1 cells were measured by western blotting (*n* = 5). J) Nuclear translocation of NRF2 (red) in THP‐1 cells was detected by immunofluorescence assay. Scale bar = 20 µm. K) Schematic illustration of the mechanism of effect of the 4‐OI targeting macrophage ARID3A to regulate NETosis through the THBS1/CD47 axis in IR injury after HT. ns: No significant, ^*^ *p* <0.05, ^**^ *p* <0.01, ^***^ *p* <0.001, ^****^ *p* <0.0001.

Our study has demonstrated that the transcription factor ARID3A plays a critical role in regulating NETs formation and myocardial injury during HT. To further determine whether ARID3A is a direct pharmacological target of 4‐OI, we performed molecular docking simulations to investigate the binding mode of the ARID3A‐4‐OI complex. In this docking pocket, 4‐OI formed a hydrogen bond at Ile342, alkyl interactions at Ala311, and Trp294, suggesting that ARID3A was a direct pharmacological target of 4‐OI (**Figure** **9**A). To validate the binding affinity of 4‐OI to ARID3A, we further performed a Surface plasmon resonance (SPR) assay. The result indicated 4‐OI has a strong binding affinity to ARID3A protein, and the binding signal increased with a higher ARID3A concentration (Figure 9B). Importantly, the elevated expression of ARID3A in macrophages induced by H/R supernatant was progressively suppressed with increasing concentrations of 4‐OI (Figure 9C). The level of THBS1 secreted by macrophages was also significantly decreased after 4‐OI treatment (Figure S12A, B, Supporting Information). Furthermore, we found that ARID3A knockdown did not significantly enhance the therapeutic effect of 4‐OI on myocardial IR injury during HT (Figure 9D–G), while NETs generation was not further decreased (Figure 9H), suggesting that 4‐OI may exert its cardioprotective effects primarily through ARID3A‐dependent mechanisms. Moreover, overexpression of ARID3A in THP‐1 cells was found to reverse the inhibitory effect of 4‐OI on NETosis by increasing THBS1 expression (Figure 9I; Figure S12C,D, Supporting Information).

Emerging evidence has demonstrated that 4‐OI could play a cardioprotective role by activating the nuclear erythroid 2‐related factor 2 (NRF2) signaling pathway, which promotes macrophage polarization and reduces inflammation.^[^ ^36^ ^]^ We next assessed whether treatment with exogenous 4‐OI induces NRF2 activation in THP‐1 cells during H/R. Interestingly, protein expression of NRF2 and heme oxygenase‐1 (HO‐1) was increased in the H/R + 4‐OI group, while overexpression of ARID3A reversed the activation of the NRF2/HO‐1 pathway by 4‐OI (Figure 9I). In addition, immunofluorescence showed that 4‐OI increased nuclear translocation of NRF2, but overexpression of ARID3A significantly decreased nuclear translocation of NRF2 (Figure 9J). In conclusion, the above results suggest that the mechanism by which 4‐OI exerts cardioprotective effects through targeting inhibition of ARID3A is not only related to limiting the activation of the THBS1/CD47 pathway, but also to the accumulation of the antioxidant factor NRF2 in macrophages (Figure 9K).

## 3 Discussion

Our work demonstrated that NETosis is one of the pathogenic mechanisms of myocardial IR injury after HT for the first time. In this study, we elucidated the characterization of non‐cardiomyocytes in transplanted hearts and further analyzed the heterogeneity of neutrophil subpopulations and macrophage subpopulations using scRNA‐seq. Furthermore, based on the characteristics of various neutrophil clusters, we identified a subpopulation of NETs‐associated neutrophils that could be potential targets for early intervention in myocardial IR injury after HT. Our findings also highlighted a putative interaction between M1 macrophages and NETs‐related neutrophils, reinforcing the notion that their crosstalk may be a viable therapeutic target in IR injury. We combined scRNA‐seq results to systematically characterize the cellular and molecular changes in NETs‐associated neutrophils and M1 macrophages following myocardial IR injury after HT. Mechanistically, we discovered that upregulation of ARID3A in M1 macrophages following HT transcriptionally activates THBS1, a secreted protein that binds to CD47 on neutrophils, thereby promoting p38 MAPK signaling and enhancing NETosis, ultimately exacerbating myocardial IR injury. Moreover, we uncovered that treatment with the 4‐OI ameliorates myocardial IR injury in vivo and protects cardiomyocytes from H/R injury in vitro. These results suggest the therapeutic potential of 4‐OI in preventing myocardial IR injury after HT. Collectively, our findings offer novel mechanistic insights and therapeutic strategies for regulating NETosis and mitigating myocardial IR injury during HT.

Although the underlying mechanisms of myocardial IR injury after HT are not fully elucidated, previous studies have implicated neutrophil‐mediated inflammatory responses as a major contributing factor.^[^ ^26a^ ^]^ However, the full spectrum of neutrophil functions in this context remains poorly characterized. In our study, we focused on neutrophils in transplanted hearts, demonstrating that their activation exacerbates the inflammatory milieu via NETosis. These structures accelerate reactive oxygen species (ROS) generation and stimulate the production of pro‐inflammatory cytokines and chemokines.^[^ ^37^ ^]^ Consistent with previous studies on myocardial warm IR injury,^[^ ^38^ ^]^ the present study demonstrated for the first time, using an ectopic HT model, that NETs are also involved in myocardial cold IR injury and that myocardial IR injury can be significantly attenuated by the use of DNase I, an enzyme degrading NETs. Thus, NETosis plays an unquestionably critical role in both myocardial warm IR injury and myocardial cold IR injury, and it is crucial to seek an effective way to inhibit NETs formation.

Neutrophils could be activated by signals from other cells via intercellular crosstalk. A comprehensive understanding of neutrophils heterogeneity and the interaction between neutrophils and other cells in the microenvironment of the transplanted hearts could allow the development of new therapeutic approaches for treating NETosis. In this study, we employed scRNA‐seq to comprehensively characterize the landscape of non‐cardiomyocyte populations in transplanted hearts and uncovered previously unrecognized interactions between macrophages and neutrophils at single‐cell resolution. Specifically, we identified 6 distinct neutrophil clusters and systematically delineated their heterogeneity and functional characteristics. Furthermore, we innovatively defined Ero1l^+^ neutrophils as a neutrophil subpopulation highly correlated with the NETs phenotype by NETs scoring and KEGG enrichment analysis, which was validated by multicolor immunofluorescence staining. Due to the different models applied, we differed from another study on myocardial IR injury in the neutrophil subpopulations identified, but consistently both confirmed the fact that NETs are present in myocardial IR injury, emphasizing their potential as diagnostic biomarkers and therapeutic targets for myocardial IR injury management.^[^ ^39^ ^]^ Given the nonspecific and proteolytic nature of the enzymes released in NETs, excessive NETosis can trigger an uncontrolled inflammatory response, promoting further neutrophil recruitment and exacerbating histopathological myocardial damage.^[^ ^10a^ ^]^ The NETs‐associated neutrophils from transplanted hearts identified in our present study indeed exhibited strong pro‐inflammatory and neutrophil chemotaxis properties as well as marked inflammatory response. Ligand‐receptor linkage analysis of the NETs‐associated neutrophil signaling network highlighted that macrophages are the primary cellular targets of NETs‐associated neutrophils. It is now widely accepted that the M1 macrophage population predominates during the early phase of myocardial IR, followed by a progressive increase in the M2 subset.^[^ ^40^ ^]^ M1 macrophages exacerbate the acute inflammatory response after IR injury by secreting pro‐inflammatory mediators and recruiting additional neutrophils, thereby amplifying local inflammation and myocardial tissue destruction.^[^ ^41^ ^]^ Although several studies on neutrophil‐macrophage interactions have been reported recently, they focused mainly on NETs regulating macrophages.^[^ ^42^ ^]^ Unlike their focus, our work validated that M1 macrophages also promote NETs production from multiple aspects, including single‐cell transcriptomics, immunofluorescent labeling, and cell co‐culture experiments. A distinguishing feature of cardiac allograft macrophages is their dual origins, with donor‐derived macrophages transplanted with the grafts and recipient monocyte‐derived macrophages subsequently recruited to transplanted hearts. In healthy hearts, monocyte‐derived macrophages are sparse, but in response to injury, they are recruited in large numbers and differentiate into functionally distinct macrophage subsets. The initial wave of infiltrating monocyte‐derived macrophages following injury are proinflammatory,^[^ ^43^ ^]^ which is also well demonstrated in our present study. In contrast to resident macrophages, which display a reparative gene expression profile, these recruited macrophages exhibit a distinct transcriptional profile and display proinflammatory properties.^[^ ^44^ ^]^ Based on our study, the application of Clo‐Lip to deplete mononuclear phagocytes in recipient mice significantly reduced the level of NETs in the transplanted hearts. These findings further confirm that monocyte‐derived macrophages recruited from the recipient play a dominant role in mediating NETosis during the early reperfusion phase of HT.

By tracing the interactions between M1 macrophages and Ero1l⁺ neutrophils, we found that THBS1, predominantly expressed by M1 macrophages, interacts with CD47 receptors on neutrophils. We therefore hypothesized that THBS1 may promote the generation of NETs in Ero1l^+^ neutrophils through ligand‐receptor signaling. THBS1 belongs to the family of extracellular matrix thrombin‐sensitive proteins and was first identified in platelets, where it forms homotrimers and binds to the cell‐surface receptors CD36 and CD47 to exert various biological effects.^[^ ^45^ ^]^ In cardiac allografts, elevated THBS1 in the graft correlates with the development of cardiac allograft vasculopathy.^[^ ^46^ ^]^ Furthermore, induction of THBS1 in renal IR injury is deleterious and could lead to tubular injury and renal failure.^[^ ^47^ ^]^ Notably, it has been reported that macrophages are the primary source of THBS1 during myocardial injury,^[^ ^48^ ^]^ and increased THBS1 could lead to increased inflammatory responses and tissue damage by stimulating excessive neutrophil recruitment and activation.^[^ ^49^ ^]^ In line with these findings, our study demonstrated that THBS1 expression in transplanted hearts is primarily derived from recruited macrophages and that exogenous THBS1 administration exacerbates neutrophil infiltration and myocardial IR injury after HT. In vitro experiments showed that H/R supernatants‐stimulated macrophages can increase THBS1 expression and secretion. Furthermore, macrophage‐derived THBS1 was shown to be a critical mediator of NETs release from neutrophils following myocardial IR, supporting previous reports that THBS1 acts as a molecular bridge facilitating macrophage‐neutrophil communication.^[^ ^50^ ^]^ As a transmembrane protein, CD47 belongs to the immunoglobulin superfamily and is commonly expressed in human cells.^[^ ^51^ ^]^ Our results showed that in vitro silencing of CD47 significantly inhibited the ability of neutrophils to release NETs. Mitogen‐activated protein kinases include extracellular signal‑regulated kinase (ERK), p38, and JNK MAPK subfamilies, which have been shown to be involved in the regulation of NETs formation through mechanisms such as increasing ROS levels.^[^ ^12^, ^52^ ^]^ Consistent with existing literature, our cellular experiments demonstrated that CD47 promotes NETs formation by activating the p38 MAPK signaling pathway. In conclusion, our study suggested that targeting the THBS1/CD47 ligand‐receptor pair has shown promising therapeutic potential in myocardial IR injury after HT.

The transcription factor ARID3A plays an important regulatory role in various cancers,^[^ ^53^ ^]^ renal fibrosis,^[^ ^54^ ^]^ systemic lupus erythematosus and primary biliary cholangitis,^[^ ^55^ ^]^ but its role in cardiovascular diseases was rarely reported. A recent study found that nuclear translocation of ARID3A could enhance macrophage glycolytic reprogramming by activating enolase 2 (ENO2) transcription.^[^ ^56^ ^]^ Another study showed that ARID3A‐upregulated macrophages exhibit a pro‐inflammatory phenotype, whereas ARID3A‐deficient macrophages exhibit a more reparative phenotype and significantly attenuate cholestatic liver injury.^[^ ^22^ ^]^ In addition, it was reported that knockdown of ARID3A in monocyte‐derived macrophages resulted in decreased expression of pro‐inflammatory cytokines and pro‐fibrotic cytokines.^[^ ^55b^ ^]^ Consistent with these findings, we demonstrated in in vitro and in vivo experiments that myocardial IR injury‐induced M1 macrophages showed high expression levels of ARID3A, and found that pro‐inflammatory cytokines were significantly reduced in transplanted hearts of mice after myeloid‐specific knockdown of ARID3A. Furthermore, we found that myeloid‐specific deletion of ARID3A attenuates myocardial IR injury and NETosis after HT by targeting THBS1 for the first time. In this study, we deeply explored the mechanism by which ARID3A regulates THBS1, and demonstrated that ARID3A undergoes nuclear translocation in myocardial IR injury‐activated macrophages and activates transcription by binding to a specific region of the THBS1 promoter.

‌ Itaconate is a pivotal immunometabolite with multifaceted roles in modulating host‐pathogen interactions, cellular activation, and intercellular communication.^[^ ^57^ ^]^ It has been demonstrated that hyperinflammation‐induced cytokine storms could be prevented by triggering endogenous itaconate accumulation in activated macrophages.^[^ ^58^ ^]^ Moreover, other report has also indicated that itaconate exerts anti‐inflammatory effects when administered in vitro and in vivo during macrophage activation and IR injury.^[^ ^59^ ^]^ Previous research has demonstrated that itaconate activates NRF2, leading to the suppression of pro‐inflammatory cytokine synthesis in macrophages. As a result of itaconate‐driven post‐translational modifications, NRF2 is released and translocated to the nucleus, where it initiates the transcription of antioxidant genes, such as NADPH quinone oxidoreductase 1, glutathione, and HO‐1.^[^ ^60^ ^]^ Here, we found that 4‐OI attenuates myocardial IR injury after HT. The mechanism involves inhibition of NETs production and M1 macrophage activation, which is consistent with previously reported findings.^[^ ^61^ ^]^ Notably, we did detect activation of NRF2 in macrophages treated with 4‐OI, as evidenced by increased nuclear translocation and elevated HO‐1 protein levels. Our study indicated a novel aspect that 4‐OI targets inhibition of ARID3A signaling in macrophages, thereby affecting transcriptional activation of THBS1 and NRF2 accumulation. Collectively, our findings suggested that itaconate and its derivatives may be a potential therapeutic approach for myocardial IR injury after HT.

Of note, there are some limitations in this study. First, although the present study revealed the therapeutic value of macrophage ARID3A and its regulated THBS1/CD47 signaling for the treatment of myocardial IR injury after HT in a mouse model, we were unable to validate it in human subjects. Second, insufficient in‐depth analysis of M2 macrophage subpopulations is also one of the limitations of this study, as the UMAP plots showed the existence of two distinct and separate subpopulations of M2 macrophages, suggesting that they may play different roles during HT. Third, the lack of specificity in using anti‐Ly6G to deplete all neutrophils in vivo limits the direct validation of the THBS1/CD47 pathway on Ero11⁺ neutrophils. The relationship between macrophages and neutrophils during myocardial IR after HT needs to be further explored in future studies.

In conclusion, the present study demonstrated that macrophage ARID3A induces NETs generation and myocardial IR injury after HT by regulating the THBS1/CD47 axis and provided strong evidence for the protective effect of 4‐OI against myocardial IR injury in HT.

## 4 Experimental Section

### Patients and Specimens

This study was performed in accordance with the principles of the Declaration of Helsinki. Peripheral blood was collected from patients who underwent HT from May 2024 to May 2025, and 15 mL of peripheral blood from healthy volunteers was collected (Table S4, Supporting Information). All participants provided informed consent prior to enrollment, and the study protocol was approved by the Ethics Committee of Renmin Hospital of Wuhan University (WDRY2024‐K140).

### Animals

C57BL/6J male mice were obtained from Hunan SJA Laboratory Animal Company. Myeloid‐specific ARID3A knockout mice (abbreviated as ARID3A^cKO^) were generated by breeding ARID3A^flox/flox^ mice with Csf1r‐Cre mice from Syagen Biotechnology Co., Ltd (Suzhou, China). ARID3A^flox/flox^ (abbreviated as ARID3A^fl/fl^) mice were utilized as controls. Mice were genotyped as described previously.^[^ ^62^ ^]^ All mice were maintained in a specific pathogen‐free facility at 22–25 °C under a light‐dark cycle with free access to a standard rodent diet and water. The animal experiments were approved by the Animal Ethics Committee of Renmin Hospital of Wuhan University (Ethical Approval Number: 20240104B).

In a subset of mice, 4‐OI (50 mg kg^−1^; Sigma–Aldrich) in 40% cyclodextrin/PBS and DNase I (10 mg kg^−1^, Roche, Switzerland) were administered intraperitoneally 12 h before recipient HT and 2 h after reperfusion. The control group was injected with the same dose of vehicle. To overexpress THBS1, mice were injected with 10 µL of rTHBS1 (100 µg mL^−1^, R&D Systems) protein locally in the heart after HT. The microinjection pump was used to inject into the superficial myocardium of the heart under a microscope. The needle was inserted to a depth of ≈0.3 mm, avoiding penetration into the ventricular cavity. Recipient mice were injected with clodronate liposomes (200 µL, 5 µg µL^−1^, Liposoma BV, Amsterdam, the Netherlands) or control/empty liposomes by tail vein 24 h prior to receiving HT.

In addition, anti‐CD47 (200 µg/mouse/time, BioXcell), anti‐THBS1 (100 µg/mouse/time, BioXcell) and anti‐Ly6G (50 µg/mouse/time, BioXcell) were injected into the recipient mice via the tail vein 24 h before mice received HT and 2 h after reperfusion. Anti‐IgG (BioXcell, USA), the isotype control, was administered at the same time, with the same dose and in the same manner as the model mice to exclude the interference of non‐specific antibody epitopes. At 24 h after the first administration of neutralizing antibodies, peripheral blood was collected from the mice, and flow cytometry was performed to determine the neutrophil depletion efficiency. The mice were euthanized 24 h after reperfusion of HT, and blood and myocardial tissues were collected under sterile conditions.

### Mouse Models of Heterotopic HT

To further simulate the role of IR injury in HT in vivo, syngeneic HT was performed in C57BL/6 mice with reference to published study.^[^ ^63^ ^]^ Briefly, male mice were anesthetized with an intraperitoneal injection of 1% sodium pentobarbital (50 mg kg^−1^). HT was performed after donor hearts were stored in histidine‐tryptophan‐ketoglutarate (HTK) solution (Dr. Franz Koehler Chemie, Germany) at 4 °C for 8 h (prolonged cold ischemia: IR) or 0.5 h (minimal cold ischemia: Con). The cryopreservation time for clinical transplanted hearts was generally 4‐6 h, and preservation times longer than 4–6 h may result in delayed function or even organ failure due to ischemic damage.^[^ ^64^ ^]^ Therefore, in combination with the pre‐experimental results, to ensure the stability of the animal model as well as the reproducibility of the experiments, this study decided to choose donor heart cryopreservation for 8 h with reference to previous published studies.^[^ ^65^ ^]^ Recipient mice were anesthetized to expose the skin of the right side of the neck, and then the right external jugular vein and the right common carotid artery were freed. Specialized catheters were then inserted separately and secured with 8‐0 nylon thread ligatures. Then the aorta of the donor heart and the right common carotid artery of the recipient were rapidly connected under the cover of ice saline‐soaked gauze, and the pulmonary artery of the donor heart and the right external jugular vein of the recipient were connected. After ligating and securing the connection, vascular clips were sequentially removed to restore blood perfusion of the donor heart while rewarming with warm saline. When the coronary and pulmonary arteries of the donor heart gradually filled with blood and the donor heart resumed beating, the skin of the neck of the recipient mice was quickly sutured and the mice were kept in a constant temperature cage. Within 24 h, some of the recipients were dead or experienced a non‐beating implanted heart due to twisted anastomosis vessels and/or thrombosis. These animals were excluded from further analysis. At 24 h post‐implantation, the remaining recipient mice were euthanized for harvesting donor hearts.

The extent of cardiomyocyte damage was assessed by measuring the expression of cardiomyocyte marker enzymes in serum. cTnI (Nanjing Jiancheng Bioengineering Institute, Nanjing, China) was analyzed following the manufacturer's instructions.

### Graft Survival and Allograft Functional Analyses

At 24 h after transplantation, the activity of the transplanted hearts was assessed by visual inspection and direct neck palpation. The Stanford Cardiac Surgery Laboratory Graft Scoring System was used for scoring, ranging from 0 (no contraction), 1 (contraction barely visible or palpable), 2 (contraction with a marked decrease in intensity but coordinated contraction nevertheless; disturbed rhythm), 3 (strong, coordinated beats but with a marked decrease in intensity or rate), and 4 (strong contraction of both ventricles at a normal heart rate). Each analysis was independently assessed by two or more observers.

### Assessment of Infarct Size

At 24 h after transplantation, the heart was quickly isolated and stored at −20 °C for 30 min. The heart was cut perpendicularly to the long axis, and the slices were immersed in 1% TTC diluted in phosphate buffer at 37 °C for 20 min in the dark. The slices were subsequently fixed with 4% paraformaldehyde.

### Single‐Cell RNA Sequencing (scRNA‐seq)

After harvest, transplanted hearts were washed in ice‐cold Roswell Park Memorial Institute 1640 (RPMI 1640) medium and dissociated using Multi‐tissue dissociation kit 2 (Miltenyi, Germany) according to instructions. The cell solution was then passed through a 40 µm cell strainer to collect the non‐cardiomyocytes, centrifuged at 500 × g for 5 min, and gently suspended with red blood cell lysis buffer (Solarbio, China) for another 10 min to remove red blood cells. ScRNA‐seq libraries were prepared using Chromium Next GEM Single Cell 3′ Reagent Kits v3 (10 × Genomics). The indexed sequencing libraries were purified with SPRI beads, quantified by quantitative PCR, and then sequenced on Illumina NovaSeq 6000 with PE150 read length. The paired‐end reads were intercepted using Cutadapt software, followed by data quality control using FastQC. Reference genome alignment for cell quality control and cell cluster analysis was performed using CellRanger, the official software of 10 × Genomics, and reference genome alignment for read2 transcriptome sequences was performed using the embedded STAR software to determine gene expression levels. After obtaining expression matrix using Cellranger, low‐quality cells were filtered. First, uses PCA (Principal Component Analysis) to reduce dimension of the highly variable gene expression matrix, uses the Stochastic clustering algorithm based on a graph, and uses UMAP (Uniform Manifold Approximation and Projection) as the clustering results. After screening the characteristic expressed genes in each cluster of cells, the expression differences of marker genes in different cell subsets are shown in the form of heatmaps. Monocle2 software was used for the pseudo‐time analysis. First, the DEGs in the cluster results were screened, dimension was reduced, and a spanning tree was constructed. Then, single cells were sequenced, and finally, the pseudotime trajectory curve of optimal cell development or differentiation was fitted. RNA velocity analysis by scVelo was performed to analyze the origin of cells. The STRING database (http:// www.string‐db.org/) was used for protein interaction analysis. The scRNA‐seq data were deposited in the National Center for Biotechnology Information database (NCBI) (accession number: PRJNA1184500; release date: November 03, 2028).

### RNA Sequencing (RNA‐Seq)

Total RNA was extracted from 4‐OI and vehicle‐treated heart tissues following HT using the TRIzol (Invitrogen, CA, USA). Ribosomal RNA was removed by the Ribo‐Zero kit to enrich mRNA. Purified mRNA was fragmented into small pieces with fragment buffer at appropriate temperature. Subsequently, RNA was reverse transcribed to cDNA and sequencing libraries were constructed. Finally, cDNA libraries from each sample were sequenced on the DNBSEQ‐T7 platform. RNA‐seq data have been deposited in Gene Expression Omnibus with accession number GSE298470 (release date: May 29, 2029).

### Cardiac Flow Speckle Tomography

Mice were anesthetized after 24 h of HT, and the transplanted heart in the neck was exposed after fixation in the supine position. The blood flow was observed using a laser Doppler perfusion imager (PeriCam PSI system, Perimed, Sweden). Blood flow was calculated using blood flow analysis software PIMsoft (version 1.5, Perimed AB, Järfälla, Sweden).

### Hematoxylin and eosin (H&E) Staining

Transplanted hearts were collected, fixed in 10% neutral buffered formalin overnight, and embedded in paraffin. Tissue blocks were sectioned at 10 µm thickness and slides were deparaffinized and rehydrated. H&E staining was performed using the H&E kit (Solarbio, China) following the supplier's instructions.

### Immunofluorescence Staining

Immunofluorescence staining was performed on both myocardial tissue samples and various cells as previously described.^[^ ^65b^ ^]^ Samples were fixed in a 4% paraformaldehyde solution, permeabilized with 0.1% Triton X‐100 in PBS for 10 min, and blocked in a 5% BSA solution for 1 h at 37 °C, after which they were washed and incubated with primary antibodies against Ly6G, CitH3, CD47, iNOS, THBS1, Ero1l, NRF2, F4/80, CD86 or ARID3A overnight at 4 °C. The details of the antibodies used are listed in Table S5 (Supporting Information). Samples were then washed in PBS with 0.1% Tween 20 and incubated with the corresponding fluorophore‐conjugated mouse or rabbit secondary antibodies for 1.5 h at room temperature before being subjected to final washing in PBS and incubated with DAPI for 10 min at room temperature. Fluorescence images were captured by a confocal laser‐scanning microscope (Leica TCS, Germany) or a fluorescence microscope (Olympus, Tokyo, Japan).

### BMDMs Isolation and Differentiation Induction

To isolate BMDMs, tibiae and femurs of WT or Arid3a^cKO^ mice were collected and their bone marrow was extracted. These bone marrow cells were resuspended with complete RPMI 1640 medium containing M‐CSF (40 ng mL^−1^; PeproTech, USA) and inoculated into culture dishes after filtering with a 70 µm filter (Miltenyi, Germany), washing, and centrifugation. After 1 week, the BMDMs were harvested for polarization induction. Untreated (M0) macrophages were stimulated with LPS (100 ng mL^−1^; Sigma–Aldrich) and IFN‐γ (20 ng mL^−1^; PeproTech). After 24 h, the effects of M1 macrophages on neutrophils and the underlying molecular mechanisms were investigated by performing additional experiments.

### Cells and Treatments

THP‐1 (RRID: CVCL_0006), RAW264.7 (RRID: CVCL_0493), H9c2 (RRID: CVCL_0286), and HL‐1 (RRID: CVCL_0303) cells were obtained from the Institute of cell Biology, Chinese Academy of Sciences (Shanghai, China). All cell lines were verified to be free of any contamination and in good condition. THP‐1 cells were cultured in RPMI 1640 medium with 10% fetal bovine serum (FBS) (Gibco, Grand Island, NY, USA), 0.05 mM β‐mercaptoethanol, 100 U mL^−1^ penicillin, and 100 µg mL^−1^ streptomycin at 37 °C in a 5% CO_2_ humidified incubator. For M1 macrophage polarization, THP‐1 cells were treated with 100 ng mL^−1^ PMA (Sigma‐Aldrich, China) for 24 h, and 100 ng mL^−1^ of LPS and 20 ng mL^−1^ of IFN‐γ for another 24 h. RAW264.7, H9c2, and HL‐1 cells were cultured in Dulbecco's modified Eagle's medium (DMEM) medium with 10% FBS, 100 U mL^−1^ penicillin, and 100 µg mL^−1^ streptomycin at 37 °C in a 5% CO_2_ humidified incubator. For M1 polarization, RAW264.7 cells were treated with 100 ng mL^−1^ of LPS and 20 ng mL^−1^ of IFN‐γ for 24 h.

HL‐60 cells (RRID: CVCL_0002, Procell, Wuhan, China) were cultured in Iscove's Modified Dulbecco's Medium (IMDM) medium with 20% FBS, 100 U mL^−1^ penicillin, and 100 µg mL^−1^ streptomycin at 37 °C in a 5% CO_2_ humidified incubator. HL‐60 cells in the logarithmic growth phase were further suspended in IMDM medium containing 1.25% dimethyl sulfoxide (DMSO) and incubated for 4 d. HL‐60 differentiation was evaluated via flow cytometry analysis. Mature neutrophil differentiation reaching 80% met the requirements and could be used for the next experiment.

To establish the H/R model in vitro, H9c2 or HL‐1 cells were placed in a hypoxic incubator under hypoxic conditions (94.5% N_2_, 0.5% O_2_, 5% CO_2_) for 6 h. After that, the cells were cultured under normal conditions (21% O_2_ and 5% CO_2_) at 37 °C for 12 h. H/R supernatants were collected for the subsequent experiments. Unless stated, the H/R supernatants derived from H9c2 cells were used to stimulate macrophages for 24 h, and 125 µm 4‐OI was given 4 h before pretreatment with H/R supernatants. To validate the potential role of NETs in cardiomyocyte injury, DNase I (1 U mL^−1^, Sigma–Aldrich) was added before co‐incubation to degrade NETs in the co‐culture system.

To overexpress THBS1 or ARID3A, THP‐1 cells were infected with adenovirus encoding THBS1 or ARID3A (Vigene, Jinan, China) particles at a multiplicity of infection (MOI) of 50 for 12 h, and then incubated with fresh RPMI 1640 medium containing 10% FBS for an additional 48 h. To knock down endogenous THBS1 in vitro, THBS1 siRNA (GenePharma, Shanghai, China) was transfected into THP‐1 cells using Lipo8000 transfection reagent (Beyotime, China), according to the manufacturer's protocol.

To overexpress CD47, HL‐60 cells were infected with adenovirus encoding CD47 (Vigene, Jinan, China) particles at a MOI of 50 for 12 h, and then incubated with fresh IMDM medium containing 20% FBS for an additional 48 h. To knock down CD47 or Ero1l in vitro, HL‐60 cells were pre‐transfected with si‐CD47 or si‐Ero1l (GenePharma, Shanghai, China) using a Lipo8000 transfection reagent (Beyotime, China) according to the manufacturer's instructions. Cells were cultured for 4 h in an incubator at 37 °C, 5% CO_2_, then incubated in fresh culture medium. After 24 h, cells were analyzed to confirm transfection. To inhibit p38 MAPK, HL‐60 cells were incubated for 12 h with 10 µm SB203580, a p38 MAPK inhibitor, as described in previously published study.^[^ ^66^ ^]^

### Neutrophil Isolation and NETs Preparation

Neutrophils were isolated from healthy volunteers or heart transplant patients using the Human Peripheral Blood Neutrophil Isolation Kit (Solarbio, China), and from the peripheral blood of control or heart transplant mice using the Mouse Peripheral Blood Neutrophil Isolation Kit (TBD sciences, Tianjin, China), according to the manufacturer's instructions. The isolated neutrophils were resuspended in RPMI 1640 medium. The freshly isolated neutrophils were stimulated with PMA (100 nm, Sigma–Aldrich) for 4 h at 37 °C in a humidified 5% CO_2_ incubator to induce NETs formation. The purification of NETs was performed as previously described.^[^ ^67^ ^]^ The levels of NETs were quantified by the Quant‐iT PicoGreen dsDNA Assay Kit (Invitrogen, USA). Purified NETs were used to treat H9c2 or HL‐1 cells for 24 h and for subsequent study.

For NETs formation assays, the neutrophils were grown on coverslips pre‐coated with 0.1% Poly‐L‐Lysine. After co‐culture with other cells or corresponding treatment, SYTOX Green (Invitrogen, USA) in PBS was carefully added to the plate without disturbing the NETs. After 15 min, the plates were imaged with a fluorescence microscope (Olympus, Tokyo, Japan). Circulating free DNA (cfDNA) was quantified using the Quant‐iT PicoGreen dsDNA Assay Kit. The levels of MPO‐DNA complex were analyzed using a commercially available enzyme‐linked immunosorbent assay (ELISA) kit (Abcam, USA) according to the manufacturer's protocol.

### CCK‐8 Analysis

Cells were harvested, suspended, and seeded into 96‐well plates. After culture for 24 h, cells were incubated with 10 µL CCK‐8 solution (Solarbio, China) for 2 h. Absorbance was detected at 450 nm with a microplate reader (Perkin Elmer, USA).

### ELISA Assay

THBS1 levels were detected by ELISA using a human THBS1 or mouse THBS1 kit (YBio, Shanghai, China), and absorbance was measured using a microplate reader with a wavelength of 450 nm.

### Luciferase Reporter Assay

Wild‐type (WT) and Thbs1 promoter luciferase reporter plasmids were constructed by GenePharma (Shanghai, China). 293T cells were co‐transfected with the corresponding plasmids with Lipofectamine 2000 (Invitrogen, Carlsbad, CA, USA). To construct a luciferase reporter gene vector containing Thbs1 promoter, the full‐length Thbs1 promoter containing wild‐type or mutant‐type was respectively cloned into pGL3‐basic vectors (Genecreate, Wuhan, China), and co‐transfected with or without ARID3A overexpression vector later. After 48 h of incubation, the activities of luciferase were measured using the Dual Luciferase Reporter Assay Kit (Promega, Madison, WI, USA).

### Chromatin Immunoprecipitation (ChIP) Assay

The ChIP assay was performed according to the manufacturer's instructions (P2078, Beyotime). Briefly, crosslinking was performed with 1% formaldehyde, and the cells were lysed in SDS buffer, and sonication was used to fragment the DNA. For ChIP, a rabbit polyclonal antibody specific to ARID3A or its respective IgG isotype control was used. The primer sequences were listed in Table S6 (Supporting Information). The Thbs1 promoter sequences were quantified by PCR and analysed by 3% agarose gel electrophoresis.

### Reverse Transcriptase‐Quantitative Polymerase Chain Reaction (RT‐qPCR)

The TRIzol reagent (Takara, Tokyo, Japan) was used to isolate Total RNA according to the manufacturer's instructions. Total RNA of 1 µg was reverse transcribed using a cDNA synthesis kit (Servicebio, China). The mRNA expression levels were detected by RT‐qPCR, which was performed using SYBR Green PCR Master Mix and an RT‐qPCR system (Bio‐Rad, USA). The primer sequences were listed in Table S6 (Supporting Information).

### Co‐immunoprecipitation (Co‐IP) Assay

Co‐IP was performed using the Protein A/G Magnetic Beads IP Kit according to the manufacturer's instructions (P2180S, Beyotime). Antibody‐conjugated immunomagnetic beads were prepared by diluting the target antibody with diluent and adding it to protein A/G magnetic beads (50 µL), followed by incubation at room temperature for 30 min and removal of the supernatant from the magnetic separator. After the samples were harvested, lysed, and centrifuged, the supernatants were gently mixed with antibody‐conjugated immunomagnetic beads to prepare an immunomagnetic beads‐antibody‐antigen complex. After washing the beads three times, the complex was resuspended in 100 µL PBS and used to detect endogenous interaction between the THBS1 and CD47 by western blotting. An isotype‐specific IgG was applied as a negative control.

### Docking Simulation

The chemical structure of 4‐OI was retrieved from the PubChem Compound database (https://pubchem.ncbi.nlm.nih.gov/), while the crystallographic structure of ARID3A was acquired from the Protein Data Bank. Preprocessing of 4‐OI and ARID3A was conducted using AutoDock software (version 4.2). AutoDockTools (version 1.5.6) was employed to remove water molecules and small ligands, introduce hydrogen atoms, and analyze protein binding sites to identify potential docking pockets. Molecular docking of 4‐OI and ARID3A was executed using AutoDock Vina (version 1.1.2) with predefined spatial coordinates. Structural analysis and visualization were carried out using PyMOL (version 2.4.0).

### Surface Plasmon Resonance (SPR) Assay

SPR experiments were performed using a Biacore T200 system. Briefly, recombinant human ARID3A protein was diluted and pre‐immobilized on a CM5 sensor chip (Cytiva, USA) according to the manufacturer's instructions, and 4‐OI diluted to different concentrations was injected into the channel at a flow rate of 30 µL min^−1^. The kinetic analysis was then performed according to the kinetic analysis program, and the interaction between ARID3A and 4‐OI was analyzed by the binding curves and KD values to analyze the interaction between ARID3A protein and 4‐OI.

### Western Blotting

Total tissue and cell proteins were extracted from RIPA buffer containing a protease inhibitor cocktail (Beyotime, China), phenylmethylsulfonyl fluoride (Beyotime, China), and phosphatase inhibitor (Beyotime, China). Proteins were separated by sodium dodecyl sulfate‐polyacrylamide gel electrophoresis (SDS‐PAGE) (8%‐12%) and transferred onto polyvinylidene difluoride (PVDF) membranes. The PVDF membrane was blocked in 5% milk for 1 h and incubated with primary antibody at 4 °C overnight. The details of the antibodies used are listed in Table S5 (Supporting Information). The following day, membranes were incubated with HRP‐conjugated anti‐rabbit (1:2000, #7074, CST) or anti‐mouse antibodies (1:2000, #7076, CST) secondary antibodies for 1 h at room temperature. The blots were visualized using a biological image analysis system (Bio‐Rad, USA) and quantified using ImageJ software.

### Flow Cytometric Analysis

Mice were euthanized, and intracardiac perfusion was performed using ice‐cold Hanks' Balanced Salt Solution to wash red blood cells. Myocardium was minced and digested in Hanks' Balanced Salt Solution containing collagenase II on a magnetic stirrer at 37 °C for 45 min. Fetal bovine serum was used to stop digestion. The digested tissue was filtered by a 70 µm filter and centrifuged (1200 rpm, 5 min). The cell pellets were suspended in 1 mL ice‐cold Hanks' Balanced Salt Solution containing 4 mL red blood cell lysis buffer for 15 min. To clear the supernatant, the mixture was centrifuged at 1500 rpm for 5 min. All samples were stained for 30 min at 4 °C with the following fluorescent antibodies: anti‐Ly6G (Biolegend, 127622), anti‐CD11b (Biolegend, 101205), anti‐F4/80 (Biolegend, 111604), anti‐CD86 (Biolegend, 159208), and anti‐CD45 (Biolegend, 147714). THP‐1 cells were collected after 4‐OI treatment, and then incubated with anti‐CD206 (Biolegend, 321110) or anti‐CD11c (Biolegend, 337239). A CytoFLEX flow cytometer (Beckman Coulter, Brea, CA, USA) was used for the acquisition of all samples, and CytExpert software (version 2.4) was used for subsequent analysis.

### Statistical Analysis

Statistical analysis was performed using GraphPad Prism 8.0. The normality of the data distribution was assessed by the Shapiro‐Wilk test, with all results expressed as mean ± standard deviations (SD). For data conforming to assumptions of normality and homogeneity of variance, two‐group comparisons employed Student's t‐test, while multi‐group comparisons utilized one‐way analysis of variance (ANOVA) supplemented by Tukey post hoc test. If the data did not meet these criteria, the Mann‐Whitney U test and Kruskal–Wallis test were applied to two‐group and multi‐group comparisons, respectively. Spearman correlation analysis was employed for analyzing the strength and direction of the linear relationship between two variables. A *p*‐value of ≤ 0.05 was considered statistically significant (ns: no significant difference; ^*^ *p* < 0.05; ^**^ *p* < 0.01; ^***^ *p *< 0.001; ^****^ *p* < 0.0001).

## Conflict of Interest

The authors declare no conflict of interest.

## Author Contributions

H.T. and Y.X. contributed equally to this work. H.T., Y.X., and Z.X. designed and supervised the experiments and revised the manuscript. H.T. and Y.X. wrote the manuscript and analyzed the data. H.T., J.Z., Z.L., Y.Z., and Y.X. performed experiments. H.T., Q.H., Y.X., and Y.L. collected and assessed clinical samples. Z.X. and Y.X. funded this study. All authors have reviewed and approved the submitted version.

## Supporting information



Supporting Information

## Data Availability

The data that support the findings of this study are available in the supplementary material of this article.
