# Supplementary material for: Inhibition of Macrophage ARID3A Alleviates Myocardial Ischemia‐Reperfusion Injury After Heart Transplantation by Reducing THBS1/CD47 Signaling‐Mediated Neutrophil Extracellular Traps Formation
Source: Adv Sci (Weinh). 2025 Sep 6;12(44):e09952. doi: 10.1002/advs.202509952 (PMC12667509; doi:10.1002/advs.202509952)
Supplement: Supplementary file 1 — Supporting Information [file ADVS-12-e09952-s001.docx]

**Supporting Information**

**Inhibition of Macrophage ARID3A Alleviates Myocardial Ischemia-Reperfusion Injury After Heart Transplantation by Reducing THBS1/CD47 Signaling-Mediated Neutrophil Extracellular Traps Formation**

*Hao Tian, Yonghong Xiong, Junbiao Zhan,* *Zhikun Lu, Yuxi Zhang, Yan Leng, Qin Huang, Zhongyuan Xia*^*^


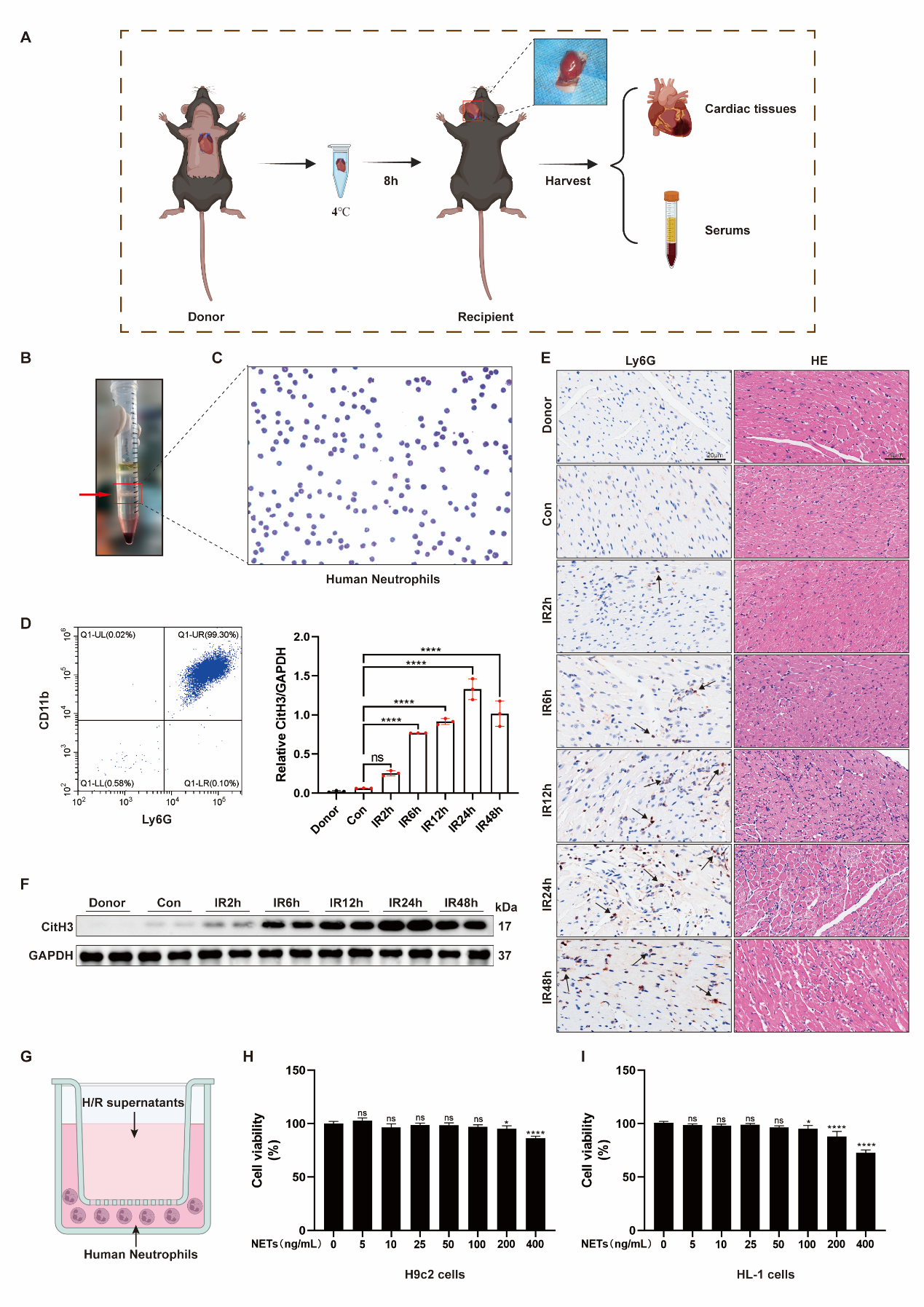


**Figure S1.** Identification of reperfusion time points for HT and mapping the concentration of NETs that stimulate cardiomyocytes. A) Graphical overview of cervical ectopic HT model in mice. B-D) Human peripheral blood neutrophils were isolated and identified for purity by Wright-Giemsa stain and flow cytometry. E) Immunohistochemistry and H&E staining were performed to detect the level of neutrophil infiltration and the degree of myocardial structural injury at different reperfusion time points. Scale bar = 20 μm. F) The NETs levels at different reperfusion time points detected by western blotting (n = 3). G) Schematic showing the co-culture system of H/R supernatants and neutrophils. H, I) The CCK-8 assay was used to determine cell viability after treatment of H9c2 and HL-1 cells with different concentrations of NETs, compared to the vehicle group (n = 5). ns: No significant, ^*^*p* < 0.05, ^**^*p* < 0.01, ^***^*p* < 0.001, ^****^*p* < 0.0001.


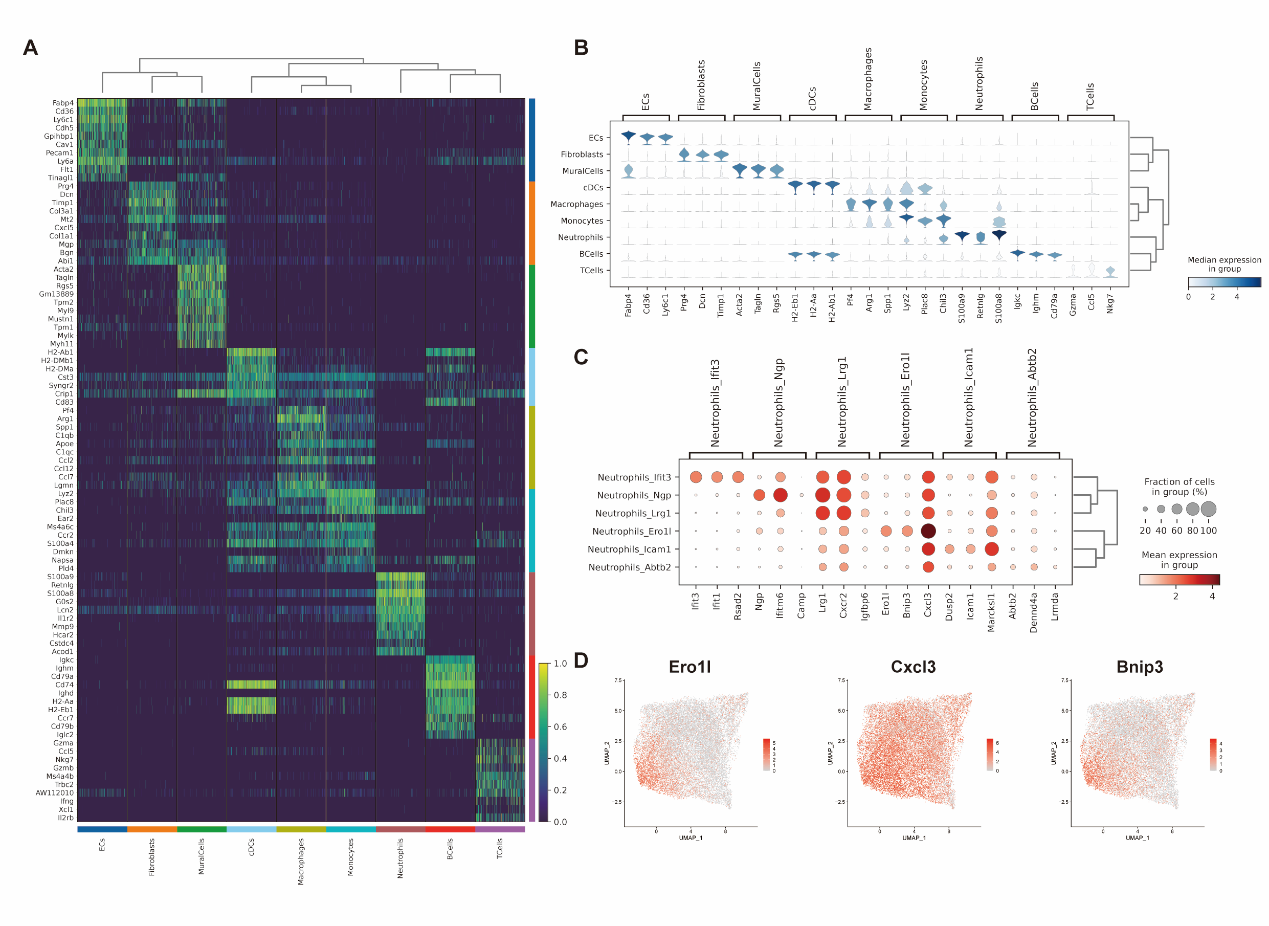


**Figure S2.** Visualization of marker genes for all cell subpopulations and neutrophil subpopulations. A) The heatmap showed the Top 10 marker genes of the 9 clusters. B) Violin plots showing the expression of marker genes for each cell type. C) Dot plots showing the expression of marker genes in each subpopulation of neutrophils. D) UMAP plots showing the expression levels of representative marker genes in Ero1l^+^ neutrophils.


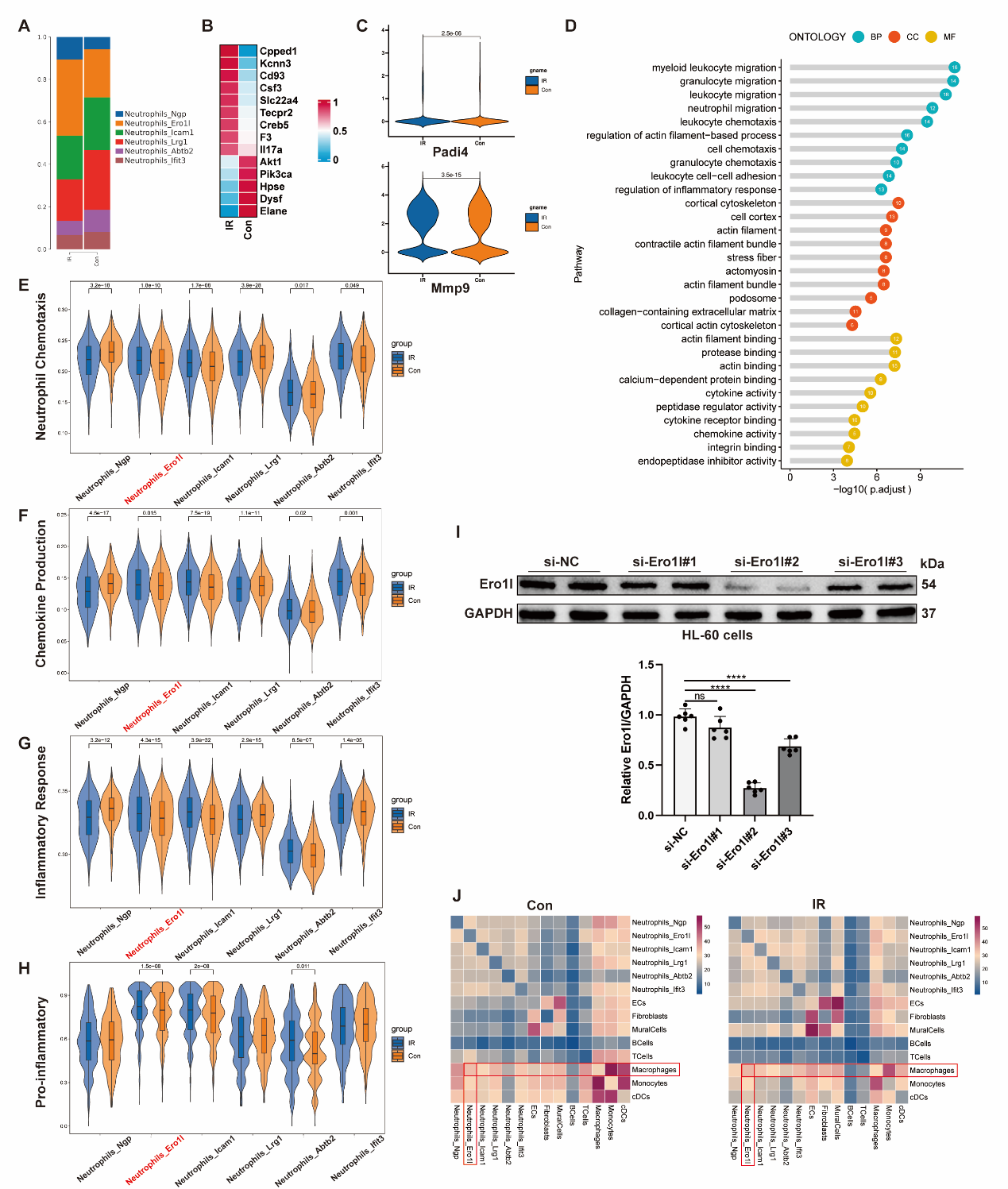


**Figure S3.** The characterization of Ero1l^+^ neutrophils. A) Bar plots show the percentage of each neutrophil subtype in scRNA-seq. B) Heatmap showing differential NETs-related gene expression in Ero1l^+^ neutrophils. C) Violin plots showing expression levels of classical NETs marker genes in Ero1l^+^ neutrophils. D) The Gene Ontology (GO) enrichment analysis of up-regulated differentially expressed genes (DEGs) in Ero1l^+^ neutrophils. E-H) Violin plots showing the scores of neutrophil chemotaxis, chemokine production, pro-inflammatory, and inflammatory response in Ero1l^+^ neutrophils. I) Ero1l protein expression level detected by western blotting proves the effect of si-Ero1l#2 transfection of HL-60 cells (n = 6). J) Heatmap showing the crosstalk between neutrophil subpopulations and other cell types. Color bars represent the number of ligand-receptor pairs. ns: No significant, ^*^*p* < 0.05, ^**^*p* < 0.01, ^***^*p* < 0.001, ^****^*p* < 0.0001.


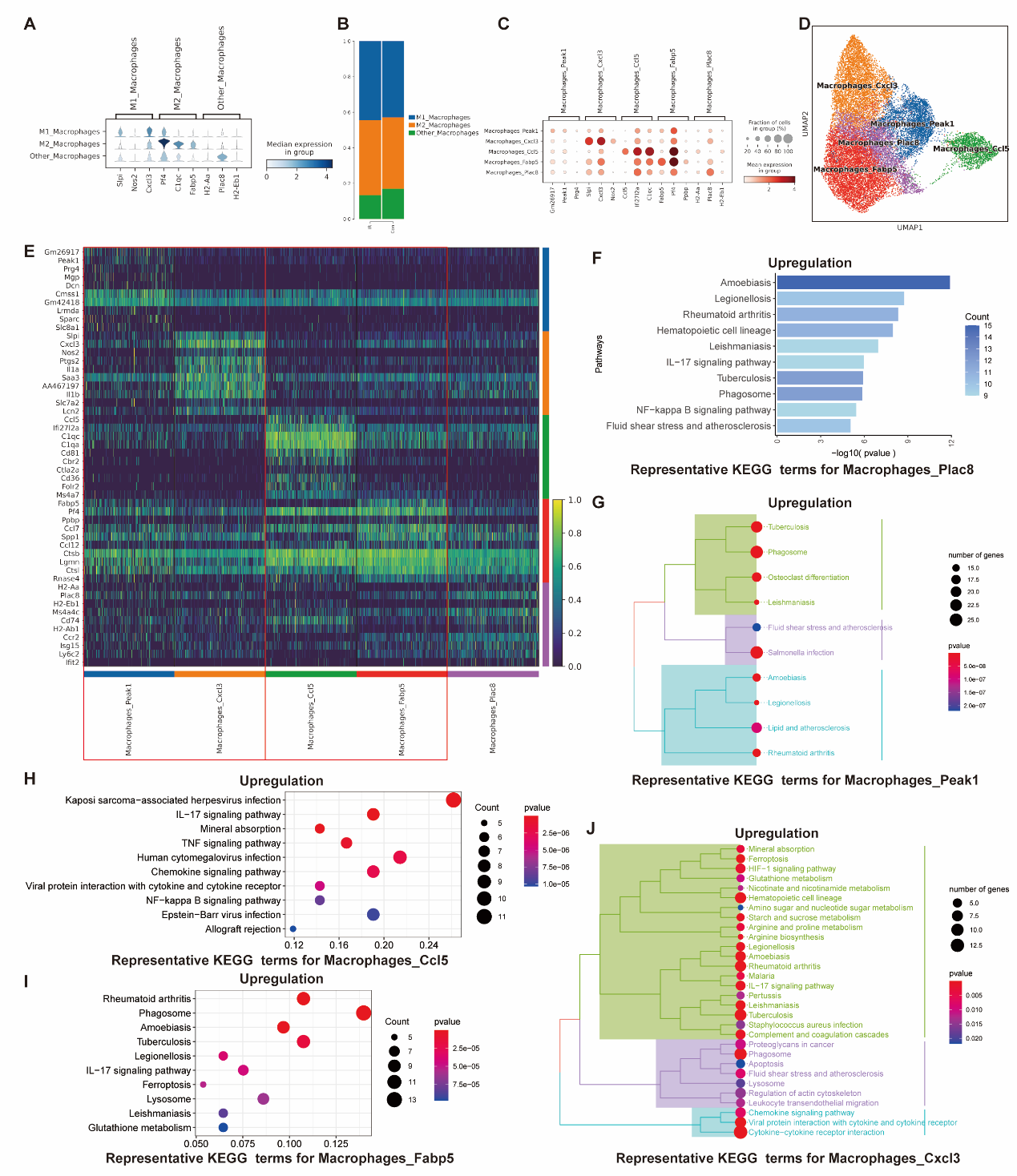


**Figure S4.** Heterogeneity and functional characterization of macrophages in myocardial IR injury during HT. A) Violin plots showing the expression levels of representative marker genes in 3 cell types. B) Bar plots showing the percentage of each macrophage subtype in scRNA-seq. C) Violin plots showing the expression levels of representative marker genes in 5 cell types. D) UMAP plots showing all macrophages clusters. E) Heatmap of differential gene expression in four macrophages. F-J) Bar charts or dot plots of KEGG enrichment analysis for each macrophage subpopulation.


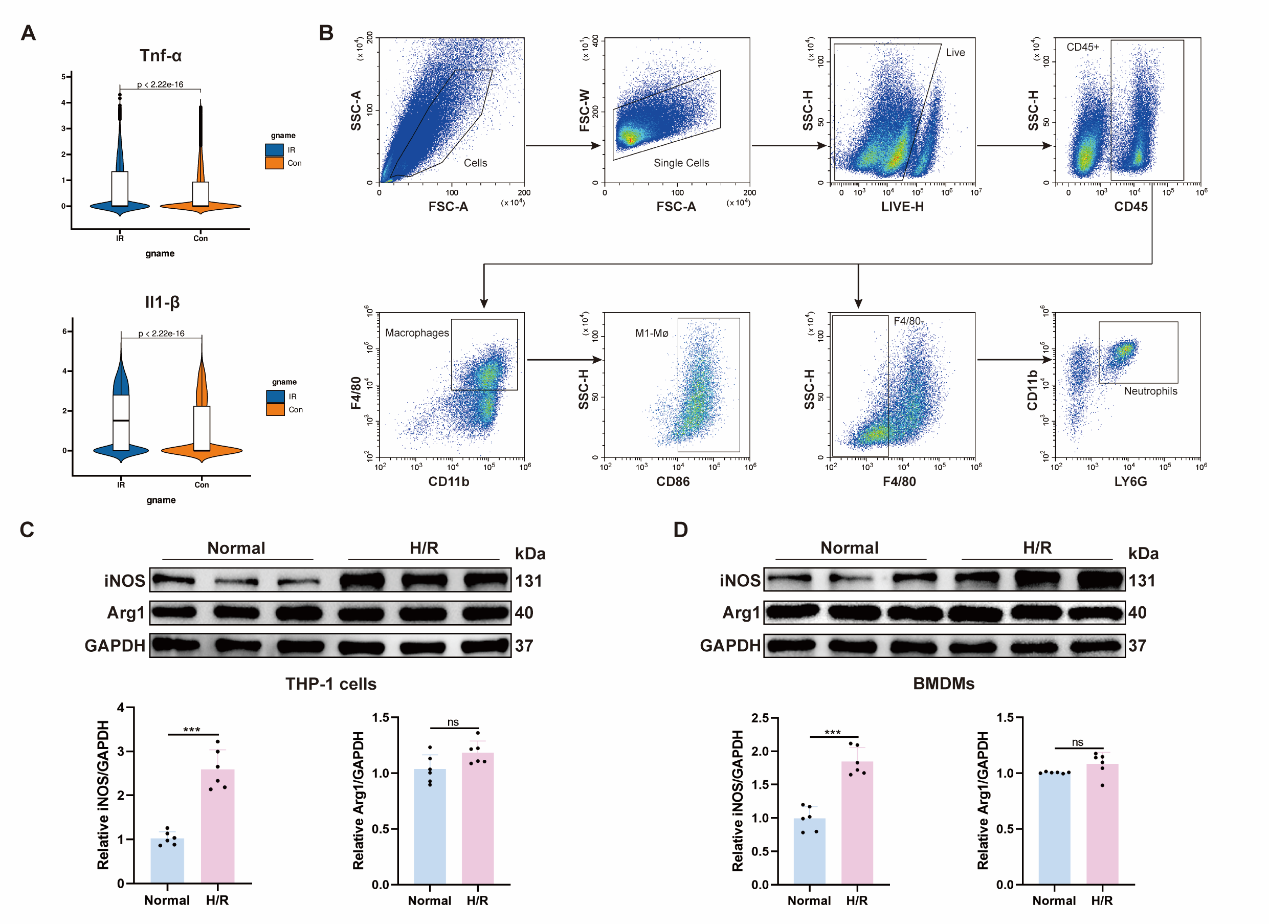


**Figure S5.** M1 macrophages are involved in myocardial IR injury after HT. A) Violin plots showing changes in expression of classical M1 marker genes in cardiac total macrophages after HT. B) The gating strategy for flow cytometry in Figure 3I. C, D) Western blotting assay for iNOS and Arg1 expression in THP-1 cells and BMDMs co-cultured with H9c2-derived H/R supernatants (n = 6). ns: No significant, ^*^*p* < 0.05, ^**^*p* < 0.01, ^***^*p* < 0.001, ^****^*p* < 0.0001.


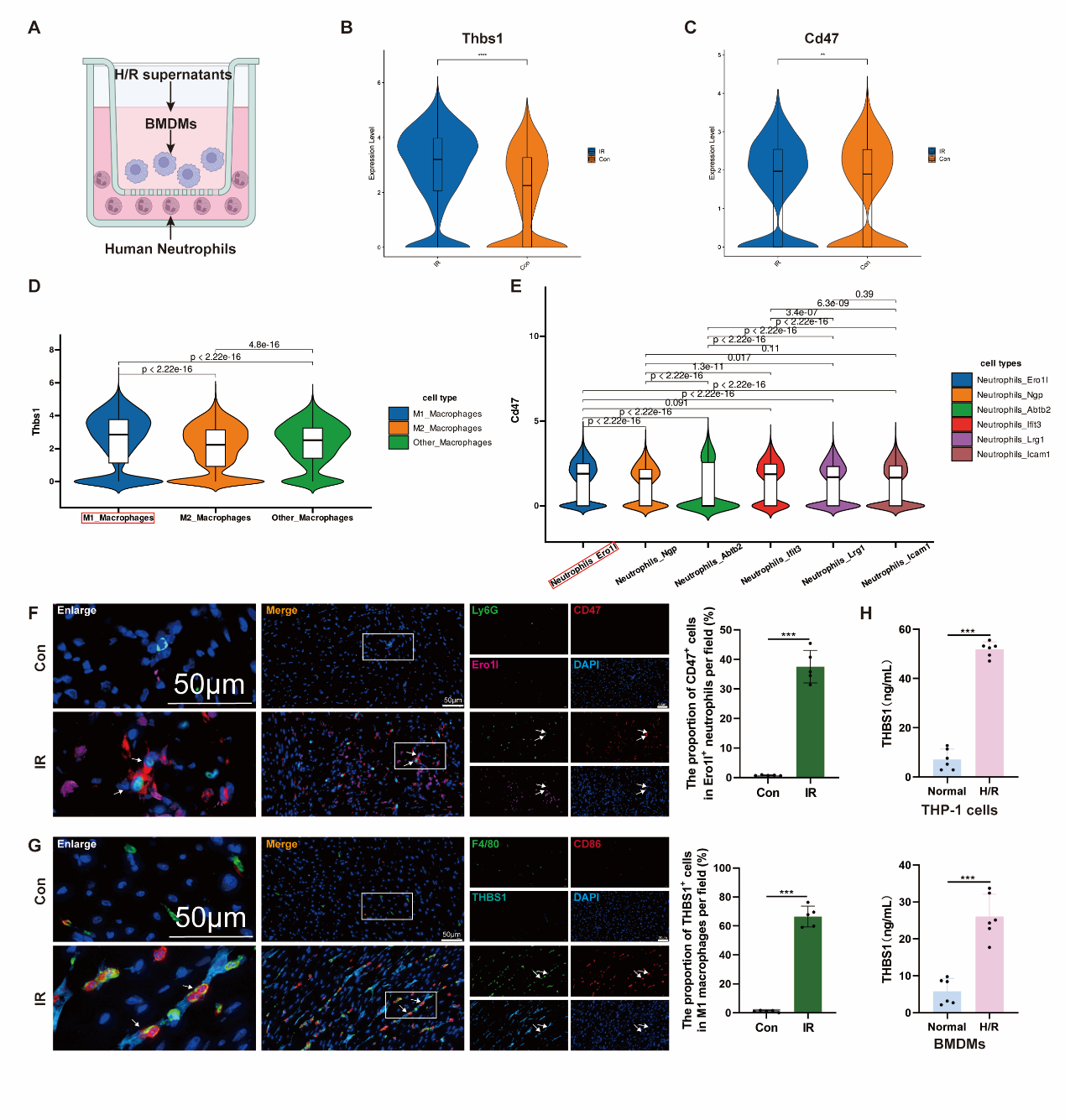


**Figure S6.** Expression levels of the THBS1/CD47 axis were increased between M1 macrophages and Ero1l^+^ neutrophils during cardiac transplant reperfusion. A) Schematic showing the co-culture system of macrophages and human peripheral blood neutrophils. B, C) Expression of Thbs1 in M1 macrophages and Cd47 in Ero1l^+^ neutrophils in scRNA-seq. D, E) The expression of Thbs1 in each macrophage subpopulation and the expression of Cd47 in each neutrophil subpopulation in the IR group. F) Representative immunofluorescence images and quantification of Ly6G^+^ CD47^+^ Ero1l^+^ cells with staining for Ly6G (green), CD47 (red), and Ero1l (magenta) in heart tissues (n = 5). Scale bar = 50 μm. G) Representative immunofluorescence images and quantification of F4/80^+^ CD86^+^ THBS1^+^ cells with staining for F4/80 (green), CD86 (red), and THBS1 (cyan) in heart tissues (n = 5). Scale bar = 50 μm. H) Quantification of THBS1 secreted by THP-1 cells and BMDMs treated or untreated with H/R supernatants (n = 6). ns: No significant, ^*^*p* < 0.05, ^**^*p* < 0.01, ^***^*p* < 0.001, ^****^*p* < 0.0001.


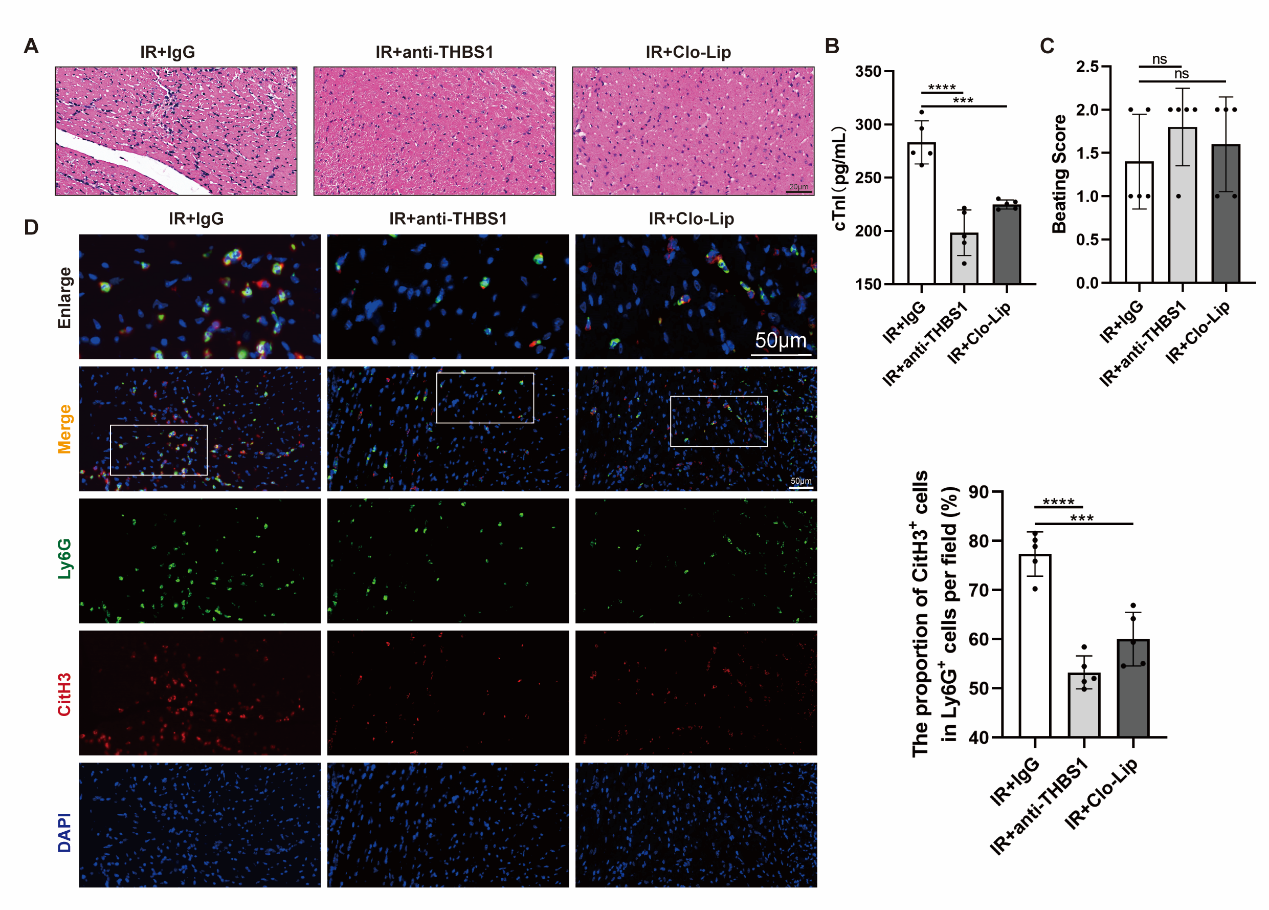


**Figure S7.** Macrophages that communicate with neutrophils after HT are recruited from recipient mice. A) Representative image of H&E staining of heart tissues. Scale bar = 20 μm. B) The serum levels of cTnI were evaluated in different groups of mice (n = 5). C) Beating score measured by the Stanford Cardiac Surgery Laboratory graft scoring system (n = 5). D) Representative immunofluorescence images and quantification of Ly6G^+^ CitH3^+^ double-positive cells with staining for Ly6G (green) and CitH3 (red) in heart tissues (n = 5). Scale bar = 50 μm. ns: No significant, ^*^*p* < 0.05, ^**^*p* < 0.01, ^***^*p* < 0.001, ^****^*p* < 0.0001.


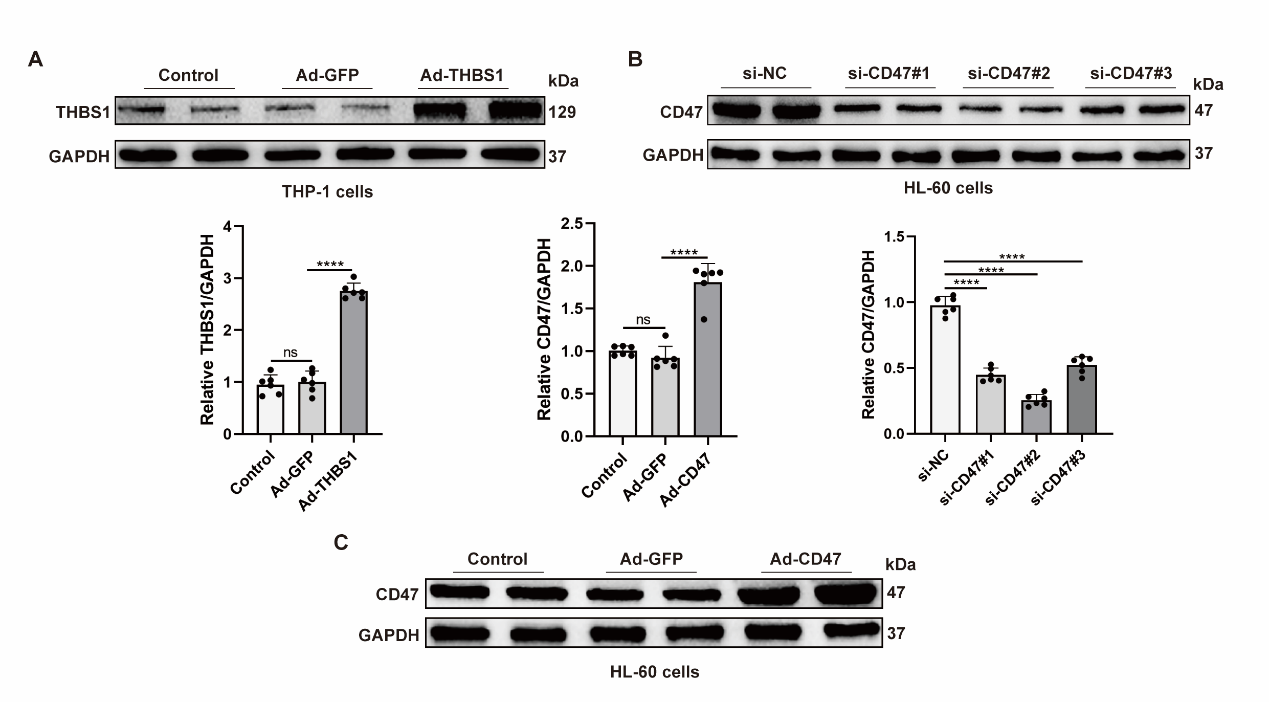


**Figure S8.** Identification of protein expression levels of THBS1 and CD47. A) THBS1 protein expression level detected by western blotting demonstrates successful adenoviral infection of THP-1 cells (n = 6). B) CD47 protein expression level detected by western blotting proves the effect of si-CD47#2 transfection of HL-60 cells (n = 6). C) CD47 protein expression level detected by western blotting proves successful adenoviral infection of HL-60 cells (n = 6). ns: No significant, ^*^*p* < 0.05, ^**^*p* < 0.01, ^***^*p* < 0.001, ^****^*p* < 0.0001.


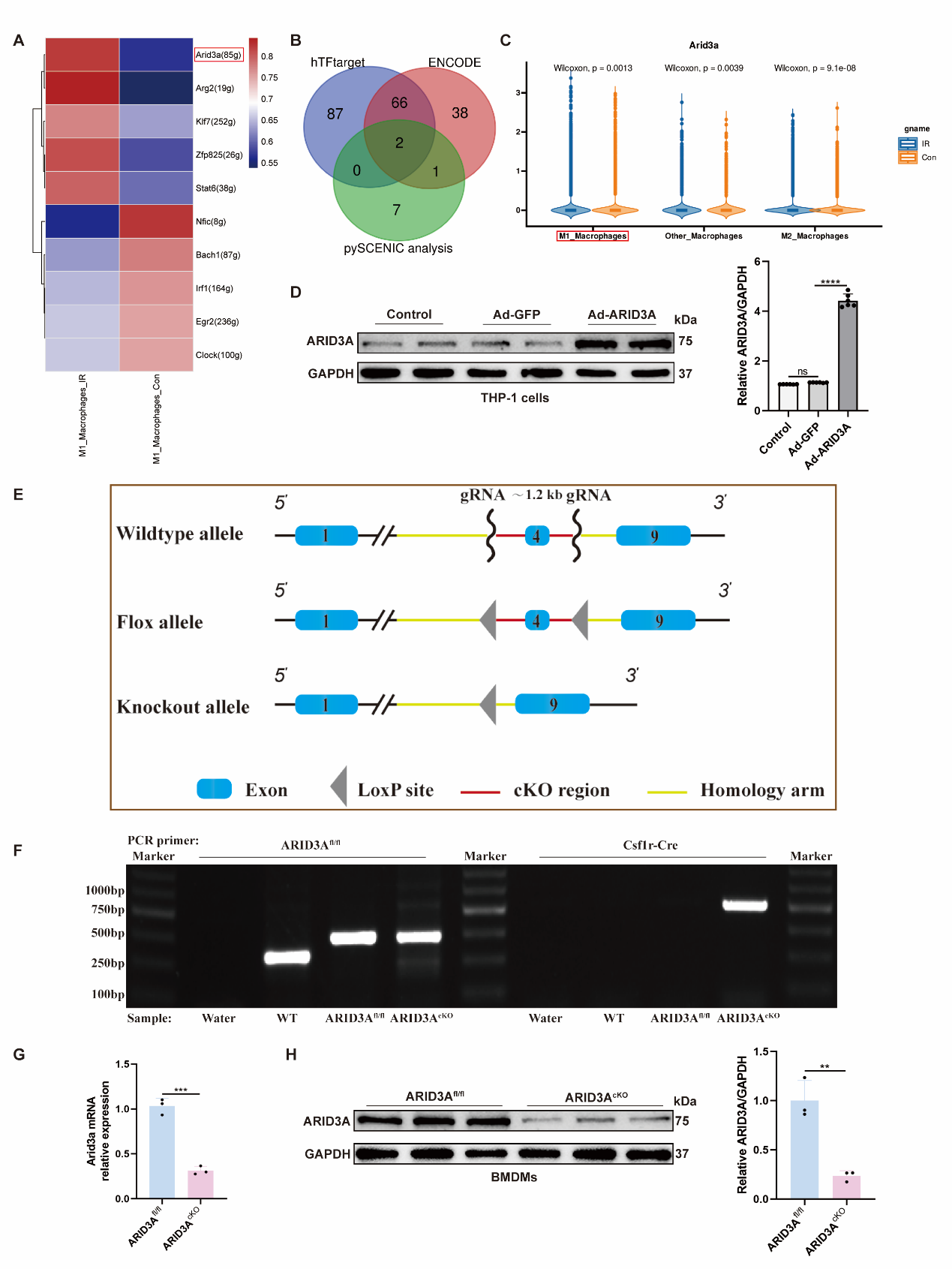


**Figure S9.** Expression of ARID3A in macrophages and identification of myeloid-specific ARID3A knockout (ARID3A^cKO^) mice. A) Heatmap showing significantly different transcription factors predicted by pySCENIC in M1 macrophages. B) Venn diagram suggested that two transcription factors (Arid3a and Nfic) were predicted by both transcription factor prediction databases and the pySCENIC analysis in this study. C) Violin plots showing the expression level of Arid3a in each macrophage subpopulation. D) ARID3A protein expression level detected by western blotting demonstrates successful adenoviral infection of THP-1 cells (n = 6). E) The schematic diagram for the construction of ARID3A^cKO^ mice. F) Representative agarose gel electrophoresis images of ARID3A^cKO^ and ARID3A^fl/fl^ mice for genotype identification. G, H) ARID3A expression level detected by western blotting and RT-qPCR indicated that Arid3a was deleted in BMDMs of ARID3A^cKO^ mice (n = 3). ns: No significant, ^*^*p* < 0.05, ^**^*p* < 0.01, ^***^*p* < 0.001, ^****^*p* < 0.0001.


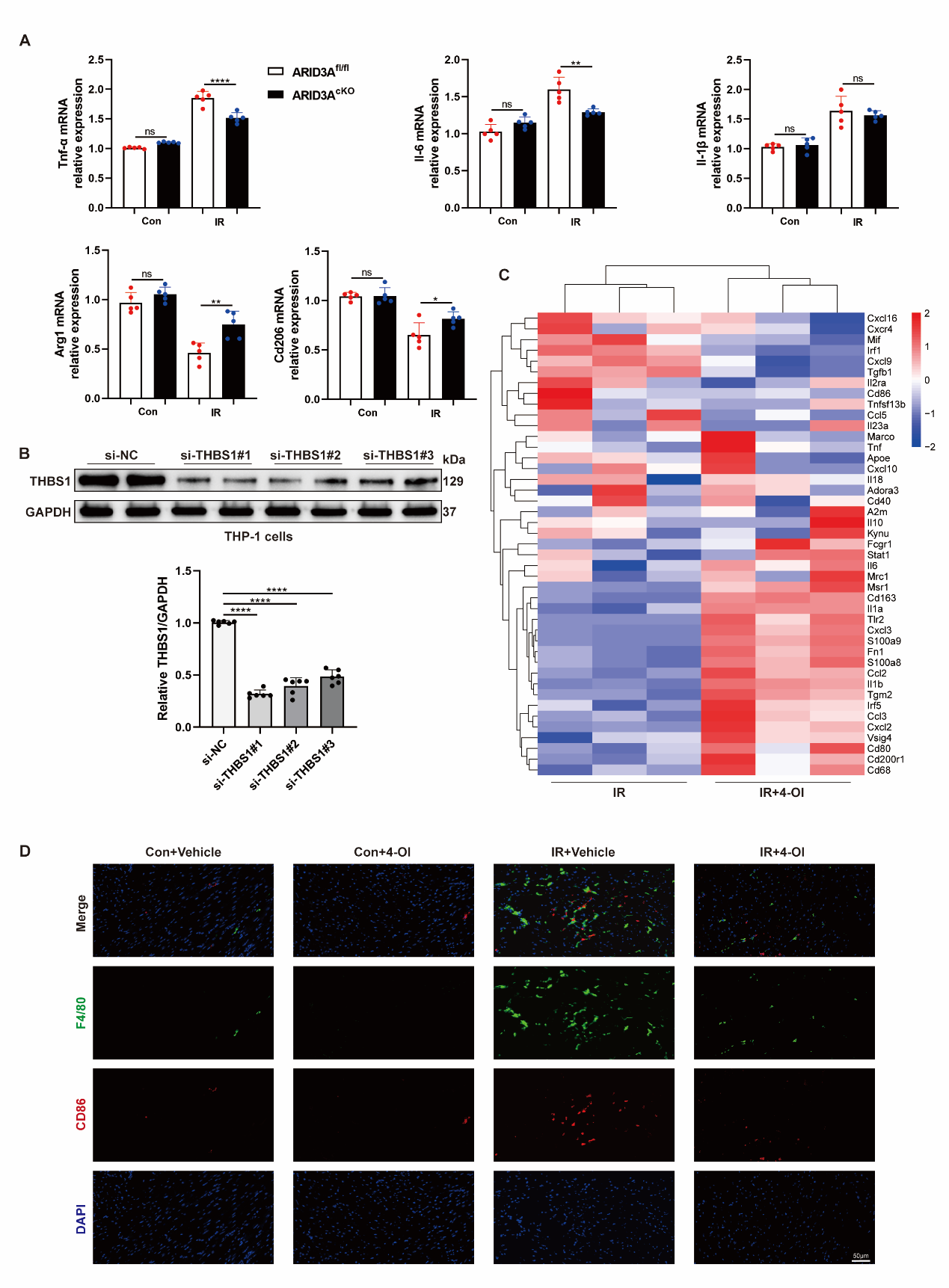


**Figure S10.** Exogenous 4-OI administration inhibits M1 macrophage polarization following myocardial IR after HT. A) Gene expression profiles of anti-inflammatory cytokines and pro-inflammatory cytokines in hearts (n = 5). B) THBS1 protein expression level detected by western blotting proves the effect of si-THBS1#1 transfection of THP-1 cells (n = 6). C) Heatmap showing different gene expression changes associated with M1 polarization and M2 polarization markers in transplanted hearts undergoing myocardial IR injury with or without 4-OI administration. D) Immunofluorescence co-staining for F4/80 (green) and CD86 (red) in the hearts of mice undergoing myocardial IR injury with or without 4-OI administration. Scale bar = 50 μm. ns: No significant, ^*^*p* < 0.05, ^**^*p* < 0.01, ^***^*p* < 0.001, ^****^*p* < 0.0001.


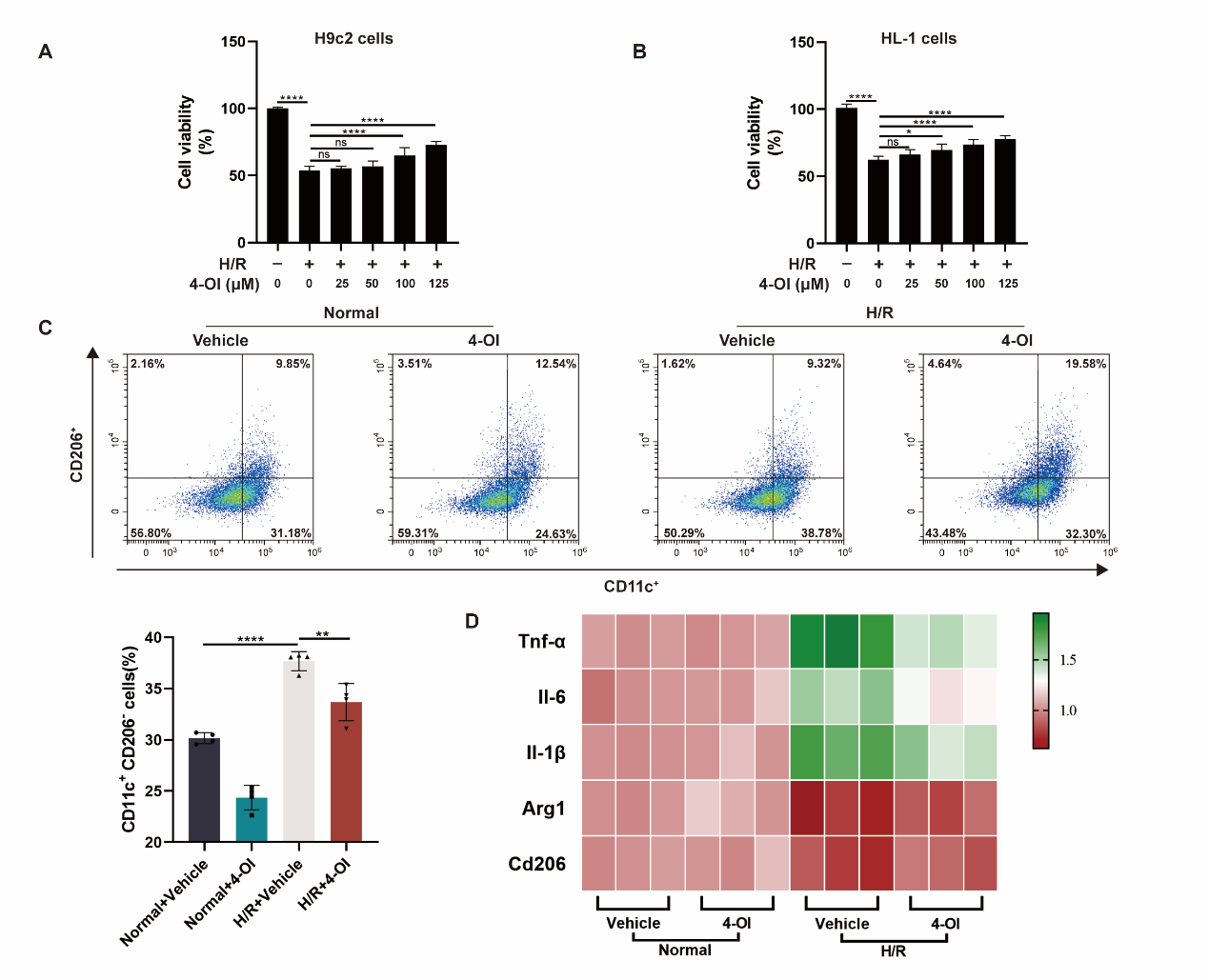


**Figure S11.** The administration of 4-OI ameliorates cardiomyocyte H/R injury by inhibiting M1 macrophage polarization in vitro. A, B) CCK-8 assay was used to determine the changes in cell viability after 4-OI administration at different concentrations to treat H9c2 and HL-1 cells (n = 6). C) Representative flow cytometry plots showing the percentage of M1 (CD11c^+^ CD206^+^) phenotype in THP-1 cells after 4-OI treatment. D) Gene expression profiles of M1 markers (Il-1β, Il-6, and Tnf-α) and M2 markers (Arg1 and Cd206) in BMDMs after 4-OI administration (n = 3). The expression of relevant genes was normalized to β-actin and expressed as relative change values. ns: No significant, ^*^*p* < 0.05, ^**^*p* < 0.01, ^***^*p* < 0.001, ^****^*p* < 0.0001.

**
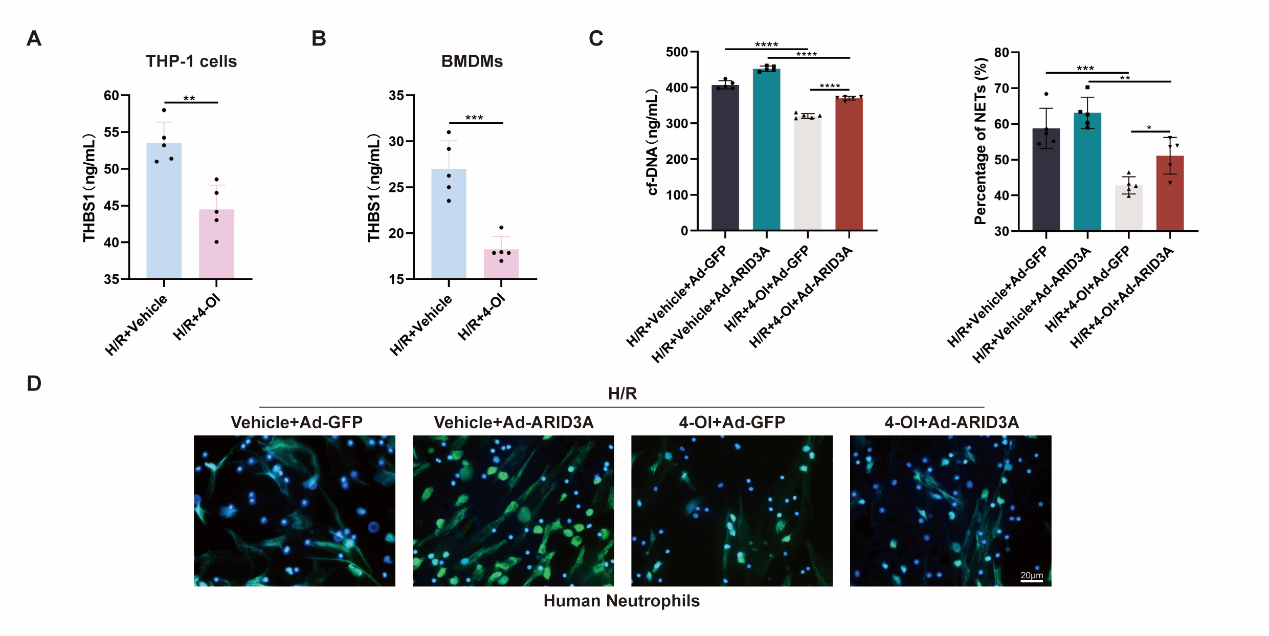
**

**Figure S12.** 4-OI reduces THBS1 secretion by macrophages and inhibits NETosis. A, B) Quantification of THBS1 secreted by THP-1 cells and BMDMs treated or untreated with 4-OI (n = 5). C) The levels of cfDNA in the supernatants of human peripheral blood neutrophils co-cultured with THP-1 cells induced by H9c2-derived H/R supernatants (n = 5). D) Images and quantitative analysis of NETs formation in human peripheral blood neutrophils co-cultured with THP-1 cells by SYTOX Green staining (n = 5). Scale bar = 20 μm. ns: No significant, ^*^*p* < 0.05, ^**^*p* < 0.01, ^***^*p* < 0.001, ^****^*p* < 0.0001.

**Table S1.** Neutrophils-associated gene set.

| **69 NETs-related genes** | Alpl, Bst1, Cd93, Ceacam3, Creb5, Crispld2, Csf3r, Cyp4f3, Dysf, Fcar, Fcgr3b, Cpped1, Fpr1, Fpr2, G0s2, Hist1h2bc, Hpse, Cxcr1, Cxcr2, Kcnj15, Lilrb2, Mgam, Mme, Pde4b, S100a12, Siglec5, Slc22a4, Slc25a37, Tecpr2, Tnfrsf10c, Vnn3, Akt1, Akt2, Atg7, Clec6a, Csf3, Ctsg, Cybb, Dnase1, Elane, Entpd4, F3, Hmgb1, Il17a, Il1b, Il6, Il8, Irak4, Itgam, Itgb2, Kcnn3, Mapk1, Mapk3, Mmp9, Mpo, Mtor, Padi4, Ptafr, Pik3ca, Ripk1, Ripk3, Selp, Selplg, Siglec14, Tlr2, Tlr4, Tlr7, Tlr8, Tnf |
| --- | --- |

**Table S2.** M1 and M2 related genes.

| **M1-related genes** | Cd68, Cd80, Cd86, Il1a, Il1b, Tnf, Il6, Il23a, Cxcl9, Cxcl10, Fcgr1, Stat1, Irf5, Ccl5, Ccl2, Cxcl3, Ccl3, Cxcr4, Tlr2, Irf1, Il18, Cxcl2, S100a8, S100a9, Cxcl16, Mif, Tnfsf13b, Cd40, Kynu |
| --- | --- |
| **M2-related genes** | Mrc1, Cd163, Adora3, Il10, Vsig4, Msr1, Tgm2, Apoe, A2m, Tgfb1, Cd200r1, Marco, Il2ra, Fn1 |

**Table S3.** Transcription factors predicted for Thbs1.

| **hTFtarget** | SPI1, CTCF, BRD4, CBFB, CEBPA, CEBPB, EGR1, ELF1, EP300, ERG, FLI1, GATA2, GATA3, GLIS1, HDAC2, KLF1, MAZ, MED1, MYB, MYH11, PAX5, PBX3, POLR2A, RAD21, RUNX1, RXRG, SMAD1, SMC3, SP1, SRF, STAG1, STAT5B, TCF12, TCF3, TFAP4, YY1, ZEB1, ETS1, ESR1, TP53, CTCFL, E2F6, GATA1, MAX, MYC, SUZ12, TEAD4, ZBTB7A, ZNF143, ZNF263, CREBBP, FOSL2, NFIC, NRF1, REST, SIN3A, TAF1, USF1, AR, ARNT, E2F1, E2F4, FOS, FOXA1, FOXM1, GABPA, JUN, KDM5B, KLF4, NRF2, PGR, RARA, SPDEF, SRC, STAT3, TCF7L2, TFAP2C, FOXA2, CTBP2, ELL2, GATAD1, GTF2B, JMJD6, JUND, NFYA, PHF8, RELA, CDK8, CDX2, E2F7, EHF, FOXG1, GMEB2, HOXA1, HOXB13, KLF5, LYL1, MECOM, MED12, RXRA, SMC1A, TAF3, TAL1, TFDP1, ZBTB7B, ZC3H8, TCF21, ATF2, CHD1, CREB1, FOXP1, HNF4A, KDM4A, NANOG, OTX2, RBBP5, SAP30, SMAD3, SMAD4, SNAI2, SP4, TAF7, USF2, LHX2, GTF2I, MAFB, ETV1, CEBPD, GRHL3, WDR5, BMI1, BRD2, RNF2, RYBP, SUMO2, TBL1X, ARID3A, BCL3, LMNB1, MAFK, MXI1, NR3C1, RB1, MYOD1, KDM4C, DUX4, KAT5, KLF9, ONECUT1, CBX8, RUNX2, GATA4, ELK3, EWSR1, PPARD/PPARG |
| --- | --- |
| **ENCODE** | ARID3A, ATF2, BACH1, BCL3, BCLAF1, BHLHE40, BRCA1, CBX3, CCNT2, CEBPB, CEBPD, CHD1, CHD2, CHD7, CREB1, CTBP2, CTCF, CTCFL, E2F4, E2F6, EBF1, EGR1, ELF1, ELK1, EP300, ESR1, EZH2, FOS, FOSL2, FOXA1, FOXA2, FOXM1, FOXP2, GABPA, GATA2, GATA3, GTF2F1, H2AFZ, HCFC1, HDAC2, HDAC6, HMGN3, HNF4A, HNF4G, JUN, JUND, KDM4A, KDM5A, KDM5B, MAFF, MAFK, MAX, MAZ, MXI1, MYBL2, MYC, MYOD1, MYOG, NANOG, NFIC, NFYA, NFYB, NR2F2, NR3C1, NRF1, PAX5, PBX3, PML, POLR2A, RAD21, RBBP5, RCOR1, REST, RFX5, RUNX3, SAP30, SIN3A, SMARCB1, SMC3, SP1, SP4, SRF, STAT1, STAT3, TAF1, TAF7, TBL1XR1, TBP, TCF12, TCF3, TCF7L2, TEAD4, TRIM28, UBTF, USF1, USF2, WRNIP1, YY1, ZBTB7A, ZC3H11A, ZEB1, ZKSCAN1, ZMIZ1, ZNF143, ZNF217, ZNF263, ZNF384 |
| **Differential TF estimated by pySCENIC analysis** | ARID3A, ARG2, KLF7, ZFP825, STAT6, NFIC, BACH1, IRF1, EGR2, CLOCK1 |

**Table S4.** The clinical characteristics of patients.

| **Sample number** | **Gender** | **Age (y)** | **Diagnosis** | **CK-MB (ng/mL)** | **cTnT (ng/L)** |
| --- | --- | --- | --- | --- | --- |
| 1 | male | 46 | Ischemic cardiomyopathy | 2.93 | 296.0 |
| 2 | male | 52 | Ischemic cardiomyopathy | 1.99 | 236.5 |
| 3 | male | 39 | Ischemic cardiomyopathy | 2.39 | 164.3 |
| 4 | male | 47 | Coronary artery disease | 1.99 | 231.6 |
| 5 | male | 44 | Ischemic cardiomyopathy | 14.36 | 363.5 |
| 6 | male | 51 | Coronary artery disease | 5.36 | 286.6 |
| 7 | male | 47 | Ischemic cardiomyopathy | 8.61 | 266.3 |

**Table S5.** Antibodies used in this study.

| **Antibody** | **Source** | **Catalog number** | **Application** |
| --- | --- | --- | --- |
| Ly6G | Abcam | ab238132 | dilution 1:100 for IF, 1:500 for IHC |
| CitH3 | Abcam | ab5103 | dilution 1:200 for IF, 1:1000 for WB |
| CD47 | ABclonl | A1838 | dilution 1:100 for IF, 1:800 for WB |
| THBS1 | Abcam | ab267388 | dilution 1:100 for IF, 1:1000 for WB |
| Ero1l | Proteintech | 12007-1-AP | dilution 1:100 for IF, 1:500 for WB |
| NRF2 | CST | #12721 | dilution 1:200 for IF, 1:1000 for WB |
| ARID3A | Abcam | ab227274 | dilution 1:100 for IF, 1:1000 for WB |
| F4/80 | Abcam | ab6640 | dilution 1:100 for IF |
| CD86 | Proteintech | 13395-1-AP | dilution 1:100 for IF, 1:1000 for WB |
| iNOS | Abcam | ab178945 | dilution 1:200 for IF, 1:1000 for WB |
| Arg1 | ABclonl | A1847 | 1:1000 for WB |
| Bax | Proteintech | 50599-2-Ig | dilution 1:2000 for WB |
| Bcl-2 | ABclonl | A20777 | dilution 1:1000 for WB |
| p38 | CST | #8690 | dilution 1:1000 for WB |
| p-p38 | CST | #4511 | dilution 1:1000 for WB |
| p-JNK | CST | #4668 | dilution 1:1000 for WB |
| JNK | CST | #9252 | dilution 1:1000 for WB |
| p-ERK | CST | #4370 | dilution 1:2000 for WB |
| ERK | CST | #4695 | dilution 1:1000 for WB |
| PAD4 | Proteintech | 17373-1-AP | dilution 1:1000 for WB |
| HO-1 | Proteintech | 10701-1-AP | dilution 1:1000 for WB |
| GAPDH | Proteintech | 60004-1-Ig | dilution 1:50000 for WB |

**Table S6.** Primer sequence in this study.

| **Gene** | **Species** | **Forward (5′→3′)** | **Reverse (5′→3′)** |
| --- | --- | --- | --- |
| Thbs1 | human | AGAAGGACTCTGACGGCGATGG | GGAATCGGCGGAAATCGGTCTC |
| Arid3a | human | CTCGTGGAGGTCATCAACAAGAAG | TATTGGGTCCGCAGGGTGAAG |
| Thbs1 | mouse | GATGGCTGGTCTCCCTGGTCTG | TCGCATCTGTTGTTGAGGCTGTC |
| Arid3a | mouse | TCAACCTGCCTACCTCCATCAC | GCCTCGCCTCTCACACTCATAG |
| Il-1β | mouse | TCGCAGCAGCACATCAACAAGAG | AGGTCCACGGGAAAGACACAGG |
| Il-6 | mouse | CTTCTTGGGACTGATGCTGGTGAC | AGGTCTGTTGGGAGTGGTATCCTC |
| Tnf-α | mouse | GCCACCACGCTCTTCTGTCTAC | GGTTTGTGAGTGTGAGGGTCTGG |
| Arg1 | mouse | GGCAACCTGTGTCCTTTCTCCTG | GGTCTACGTCTCGCAAGCCAATG |
| Cd206 | mouse | TCGGACGGATGGCTCTGGTG | TCGCTTCCCTCAAAGTGCAATGG |
| si-CD47#1 | human | CACAGGAAACUACACUUGUTT | ACAAGUGUAGUUUCCUGUGTT |
| si-CD47#2 | human | GCCUUGGUUUAAUUGUGACTT | GUCACAAUUAAACCAAGGCTT |
| si-CD47#3 | human | GCACAAUUACUUGGACUAGTT | CUAGUCCAAGUAAUUGUGCTT |
| si-THBS1#1 | human | GACCAAAGCCUGCAAGAAATT | UUUCUUGCAGGCUUUGGUCTT |
| si-THBS1#2 | human | GGAGUUCAGUACAGAAAUATT | UAUUUCUGUACUGAACUCCTT |
| si-THBS1#3 | human | CCGAAAGGGACGAUGACUATT | UAGUCAUCGUCCCUUUCGGTT |
| si-Ero1l#1 | human | GCGAGCUACAAGUAUUCUGAATT | UUCAGAAUACUUGUAGCUCGCTT |
| si-Ero1l#2 | human | GCCAGAAAGUGGACCUAGUUATT | UAACUAGGUCCACUUUCUGGCTT |
| si-Ero1l#3 | human | GCUGAAUAUGUAGAUUUGCUUTT | AAGCAAAUCUACAUAUUCAGCTT |
| Thbs1-chip-P1 | mouse | GGTCCAACCTTTTGCCCTCT | TTCCATGACAGAGACGCTTGG |
| Thbs1-chip-P2 | mouse | TCGATCCCCAAGCGTCTCT | CGCTTAGGATCTTGCGCCAT |
| ARID3A^flox/flox^ | mouse | CATCAGACAGACAGAGAAAATGGC | TGGAGTGACTGAGTGACCTTCT |
| Csf1r-Cre | mouse | CAGAAGAGGAGAGCTCCAGTGAAC | CTTCATCAGAGGTGGCATCCAC |
| Gapdh | human | GTGGACCTGACCTGCCGTCTAG | GAGTGGGTGTCGCTGTTGAAGTC |
| β-actin | mouse | GTGACGTTGACATCCGTAAAGA | GTAACAGTCCGCCTAGAAGCAC |
